# Warm needle acupuncture for osteoarthritis: An overview of systematic reviews and meta-analysis

**DOI:** 10.3389/fmed.2023.971147

**Published:** 2023-03-14

**Authors:** Ji Hee Jun, Tae-Young Choi, Sunju Park, Myeong Soo Lee

**Affiliations:** ^1^KM Science Research Division, Korea Institute of Oriental Medicine, Daejeon, Republic of Korea; ^2^Department of Preventive Medicine, College of Korean Medicine, Daejeon University, Daejeon, Republic of Korea

**Keywords:** acupuncture, moxibustion, warm needle acupuncture, osteoarthritis, overview, systematic review

## Abstract

**Background:**

Osteoarthritis (OA) is a chronic disease that is a major cause of pain and functional disability. Warm needle acupuncture (WA) therapy has been widely used to treat OA. This overview summarizes the evidence from systematic reviews (SRs) and assesses the methodological quality of previous SRs that evaluated the use of WA therapy for OA.

**Methods:**

We searched electronic databases to identify SRs that evaluated the efficacy of WA therapy for OA. Two reviewers independently extracted data and assessed the methodological quality of the reviews according to the A Measurement Tool to Assess Systematic Reviews (AMSTAR 2) tool. The reporting quality was assessed using the Preferred Reporting Items for Systematic Reviews and Meta-Analysis 2020 (PRISMA 2020) guidelines. The quality of evidence was assessed according to the Grading of Recommendations Assessment, Development, and Evaluation (GRADE) approach.

**Results:**

Fifteen SRs were included in this study. WA therapy was more effective than control conditions for the treatment of OA. The results of the AMSTAR 2 tool showed that the methodological quality of all included studies was critically low. The items with the lowest scores were item 2 (reporting the protocol), item 7 (listing excluded studies and justifying the exclusions), and item 16 (including conflicts of interest). Regarding the PRISMA guidelines, 2 SRs exhibited greater than 85% compliance. The overall quality of evidence in the included SRs ranged from “very low” to “moderate.”

**Conclusion:**

This overview shows that WA therapy was more effective than the control treatment for OA. However, the methodological quality of the reviews was low, indicating the need for improvements in the collection of evidence. Future studies are needed to collect high-quality evidence regarding the use of WA for OA.

**Systematic review registration:**

https://www.researchregistry.com/, Research Registry (reviewregistry1317).

## Introduction

Osteoarthritis (OA) is a common chronic disease and a main symptom of joint stiffness, instability, and weakness. It usually occurs in middle-aged (between 50 and 60 years of age) people, and in particular, it occurs more often among women than men (1, 2). According to research results, the costs directly incurred by OA are billions of US dollars per year (3, 4). Therefore, the treatment of OA is significant for reducing pain in patients and alleviating the socioeconomic burden.

Traditional medicine has been used for thousands of years to treat numerous diseases and has been used to relieve pain and improve the function of the knee joint in OA patients (5, 6). Acupuncture is one of the options for treating OA (7, 8). WA is one type of acupuncture combined with moxibustion (9). The heat of the needle is transmitted to the deep part of the acupoint through the needle, which helps reduce pain and improve function. Recently, the number of studies using warm needle acupuncture (WA) for the treatment of musculoskeletal pain has increased, and the quality of the studies has gradually improved (5, 10). Systematic review (SR) is performed on a particular topic in order to provide a comprehensive and unbiased clinical evidence based on rigorous studies (11). One recent SR analyzed 66 randomized controlled trials (RCTs) and showed beneficial effects of WA for OA (12).

An overview of SRs is a method for compiling evidence and synthesizing the results of various SRs (13, 14). The greater the amount of information gathered, the better the quality of evidence that can be provided for clinical work. An overview of SRs on traditional Chinese medicine (TCM) for knee OA (15) and acupuncture for knee OA has been published recently (8, 16), which concluded that TCM generally appears to be effective for the treatment of knee OA. Nevertheless, the effectiveness of WA as a treatment for OA has not been thoroughly evaluated.

The purpose of this study was to summarize the efficacy of WA in the treatment of OA presented in SRs and to evaluate the methodological quality of the SRs.

## Methods

We followed the Preferred Reporting Items for Overviews of SRs (PRIOR) statement (17). This overview was registered in the Research Registry (reviewregistry1317) (18).

### Data sources and search strategy

An electronic literature search was conducted in PubMed, the Cochrane Register of Controlled Trials (CENTRAL), Embase, three Chinese databases (CNKI, VIP, and Wanfang), and six Korean databases (Research Information Service System (RISS), the Korean Studies Information Services System (KISS), Korean Medical Database (KMbase), DBPIA, (Korean Traditional Knowledge Portal) KTKP, KoreaMed, and Oriental Medicine Advanced Searching Integrated System (OASIS)) from their inception to January 2023. The search terms were (“warm needle acupuncture” OR “wen zhen” OR “warm acupuncture” OR “warm needle moxibustion”) AND (“osteoarthritis”) AND (“systematic review” OR “Meta-analysis”) in Korean, Chinese, and English. The search terms and websites of 12 databases are described in Supplementary 1.

### Inclusion and exclusion criteria

#### Types of studies

SRs and meta-analyses of randomized controlled trials (RCTs) or quasi-RCTs that used WA for OA were included.

#### Population

Studies of participants diagnosed with OA. There were no restrictions regarding sex or age.

#### Intervention and comparators

Studies that used WA as an intervention to treat OA were included regardless of types of comparators. Moreover, studies in which WA was combined with other therapies were also included.

#### Outcomes

SRs reporting on patient health outcomes were included. The studies included data on at least one outcome evaluating the total treatment effect and clinical symptom of interest.

### Study selection and data extraction

Two reviewers (JHJ and TYC) separately assessed the citations obtained during the search, and full-text publications from potentially relevant SRs were retrieved and appraised for inclusion. One reviewer (JHJ) extracted the data using a standardized form. Two reviewers (JHJ and TYC) independently evaluated the retrieved data, and any differences were addressed through discussions between the two authors (SP and MSL) and were resolved by discussion. The data extracted from the reviews included the first author, publication year, data search, number of trials included, interventions, comparators, outcomes, direction of effect, overall risk of bias, conclusion, and adverse events. An assessment of the methodological quality for each included SR was also conducted.

### Overlap calculation of the reviews

The degree of overlap of the original literature for SRs was assessed by creating citation metrics for SRs. We calculated the “corrected covered area” (CCA) index (19, 20). The measure of overlap dividing the frequency of repeated occurrences of the index publication in other reviews by the product of index publications and reviews is reduced by the number of index publications. Calculation formulas were calculated as CCA = (N – *r*)/(*rc* -*r*), where N is the number of included publications in evidence synthesis (this is the sum of the ticked boxes in the citation matrix), *r* is the number of rows (number of index publications), and *c* is the number of columns (number of reviews) (supplement overlap). The calculation results lower than 5 can be considered a “slight overlap,” 6–10 can be considered a “moderate overlap,” 11–15 can be considered a “high overlap,” and greater than or equal to 15 can be considered a “very high overlap.”

### Methodological quality assessment

The quality of the included SRs was evaluated using the Assessing the Methodological Quality of Systematic Reviews 2 (AMSTAR 2) tool (21). There were 16 evaluation items. The reporting was assessed as being sufficiently reported and performed (Yes), insufficiently reported (Partial Yes), or not reported (No). The overall confidence in the results of the review was rated as follows: critically low quality (more than one critical flaw with or without non-critical weaknesses), low quality (one critical flaw with or without non-critical weaknesses), moderate quality (more than one non-critical weakness), and high quality (zero or one non-critical weakness). The AMSTAR 2 tool was used by two authors (JHJ and TYC). If there was a disagreement, the other authors (SP and MSL) resolved the disagreement.

### Reporting quality assessment

We used the Preferred Reporting Items for Systematic Review and Meta-Analyses (PRISMA 2020) checklist (22). There were 27 element items that evaluated SR reporting quality. “Yes,” “Partial yes,” or “NO” were used to respond to each item. We reported the results as a ratio.

### Certainty of evidence

The quality of outcomes of the included SRs was evaluated by the Grading of Recommendations Assessment, Development, and Evaluation (GRADE) tool^1^ (23, 24). If the GRADE tool was not used in SRs, we evaluated the strength of evidence from primary trials. The assessment of the included SRs was independently carried out by the reviewers. The five categories of GRADE influenced (i.e., downgraded or upgraded) the quality of evidence and included risk of bias, inconsistency, indirectness, imprecision, and publication bias. The quality of evidence of SRs was rated as “high,” “moderate,” “low,” and “very low.” Evidence based on RCTs began as high quality. Two authors (JHJ and TYC) assessed the quality of evidence. Disagreements were resolved by discussion with a third author (MSL).

### Data synthesis and analysis

Narrative synthesis was provided because of the high heterogeneity. The results of the WA intervention were also narratively summarized in more detail from the included SRs, and the direction of effects was calculated. Such a detailed form included the features of the intervention, methodological quality, and quality of evidence.

## Results

### Study selection

**Figure 1:**
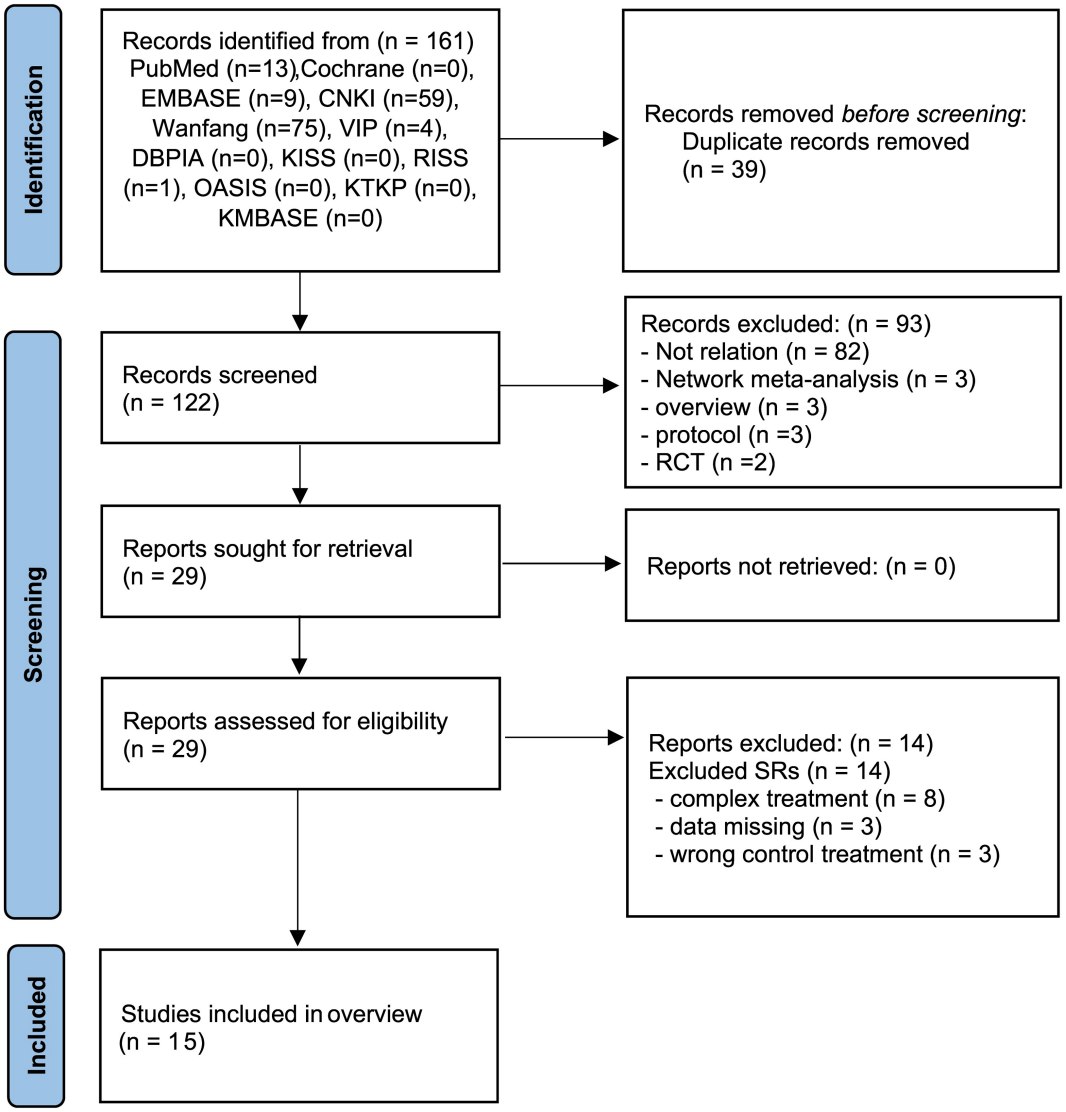
PRISMA diagram for the included studies. RCT, randomized controlled trials; SR, systematic review.

Twelve database searches identified 161 potentially relevant studies, with 39 repeated studies removed. Of the remaining 122 studies, 93 studies were excluded due to lack of relation, review, protocol, and RCT designs. A total of 29 studies were obtained after retrieval. After the final reading of the full texts, 15 SRs (12, 25–38) were included in this review. The details of the SR selection screening process are shown in Figure 1. The list of excluded studies and reasons for exclusion are shown in Supplementary 2.

### Characteristics of the included studies

Fourteen SRs (25–38) were conducted in China. They were published between 2015 and 2022, of which 40% were published in 2019. Thirteen SRs (25–33, 35–38) were published in Chinese, and two SRs (12, 34) were published in English. The SRs included between 8 and 66 primary studies. In total, the reviews included 155 different RCTs and 2 clinical control trials (CCTs). The total number of participants in the SRs was 13,940 participants. Five SRs (25, 27, 36–38) evaluated the included studies using the Jadad scale, nine SRs (12, 26, 28–30, 32–35) evaluated studies using the Cochrane risk of bias (ROB) tool, and one SR (31) did not mention an evaluation tool. All SRs conducted a meta-analysis as a statistical approach. The outcomes included in the SRs varied widely; however, they mainly focused on the overall total effective rate, visual analog scale (VAS) scores, the LKSS, and the Western Ontario and McMaster Universities Osteoarthritis Index (WOMAC). One SR (12) focused on 3 outcomes: total effective rate, pain, and function. Five SRs (12, 27, 30, 33, 35) assessed the adverse effects of WA treatment of OA. Nine SRs (25–27, 29–34, 37) arrived at a clearly positive conclusion, four SRs (12, 28, 35, 36) were neither positive nor negative, and one SR (38) drew a negative conclusion.

Various comparisons among the studies in the included SRs included WA versus Western medicine (12, 25–27), WA versus traditional medicine (acupuncture, electroacupuncture, and EA) (34–38), WA versus all types of therapies (including traditional medicine and Western medicine) (32, 33), WA plus Western medicine versus Western medicine (12, 28–30), and WA plus all types of therapies vs. all types of therapies (31). The data from the included SRs are summarized in Table 1.

**Table 1:** Summary of systematic review studies of warm needle acupuncture for knee osteoarthritis.

**First author (year) [ref]**	**Data search**	**Number of trials included (total sample size)**	**Intervention**	**Comparator**	**Outcomes**	Direction of effect*	**Overall risk of bias of primary studies**	**Methodological quality of SRs (AMSTAR 2)**	**Conclusion (Quote)**	**AEs**
Feng (2019) (25)	Jun 2018	8 (582)	WA	WM	Total effective rate	+	Jadad (High)	Critically low	… positive effective…	No
Guo (2018) (26)	Oct 2017	11 (930)	WA	WM	(1) Total effective rate	(1) +	Cochrane ROB (High)	Critically low	…effective…	No
					(2) WOMAC	(2) +				
Lu (2015) (27)	Dec 2014	8 (811)	WA	WM	(1) Total effective rate	(1) +	Jadad (High)	Critically low	…conclude be an effect...	Yes
					(2) AEs	(2) +				
Kong (2019) (28)	Mar 2018	21 (1810)	WA + WM	WM	(1) Total effective rate	(1) +	Cochrane ROB (High)	Low	… improve the efficiency…	No
					(2) Pain (VAS)	(2) +				
					(3) WOMAC	(3) +				
					(4) LKSS	(4) +				
Cao (2019) (29)	Mar 2018	9 (807)	WA + WM	WM	Total effective rate	+	Cochrane ROB (High)	Critically low	…effect…	No
Jun (2022) (12)	May 2022	66 (6231)	WA orWA + WM	WM	(1) Total effective rate	(1) +	Cochrane ROB (High)	Moderate	…have some distinct advantage…	Yes
					(2) Pain	(2) +/–				
					(3) Function	(3) +/–				
					(4) QoL	(4) +				
Jiang (2019) (30)	Jul 2018	20 (18 RCT, 2 CCT) (1719)	WA + WM	WM	Total effective rate	+	Cochrane ROB (High)	Critically low	…significant effect…	Yes
Chen (2019) (31)	Jul 2018	19 (1943)	WA orWA + WM	No limited(TDP, WM)	(1) Pain (VAS)	(1) +	n.r.	Critically low	…can increase efficacy…	No
				(2) WOMAC total	(2) +					
Huang (2021) (32)	Feb 2020	18 (1209)	WA	No limited(AT, EA, Moxa, WM)	(1) Total effective rate	(1) +	Cochrane ROB (High)	Low	…effective…	No
					(2) Pain (VAS)	(2) +				
					(3) WOMAC	(3) +				
Luo (2019) (33)	Oct 2017	10 (819)	WA	No limited (AT, EA, Moxa, WM)	Total effective rate	+	Cochrane ROB (High)	Critically low	… positive effective…	Yes
Jin (2022) (34)	Oct 2021	8 (399)	WM	TCM	(1) Total effective rate	(1) +	Cochrane ROB (High)	Low	… better overall… efficacy	No
					(2) Pain (VAS)	(2) +				
					(3) WOMAC	(3) +				
Li (2021) (35)	Dec 2019	17 (1515)	WA	AT or EA	(1) Total effective rate	(1) +/–	Cochrane ROB (High)	Low	…superior to EA…	Yes
					(2) Pain (VAS)	(2) –				
					(3) WOMAC	(3) +/–				
					(4) LKSS	(4) –				
Zhang (2018) (36)	Jun 2018	12 (1176)	WA	AT or EA	(1) Total effective rate	(1) +	Jadad (High)	Critically low	…no…significant difference…	No
					(2) Pain (VAS)	(2)–				
					(3) WOMAC	(3) –				
					(4) LKSS	(4) +				
Ou (2018) (37)	Jul 2017	7 (530)	WA	AT	Total effective rate	+	Jadad (High)	Critically low	… positive effective…	No
Wu (2016) (38)	Nov 2014	11 (772)	WA	EA	(1) Total effective rate	(1)–	Jadad (High)	Critically low	… is not superior to that of EA...	No
					(2) Pain (VAS)	(2)–				
					(3) WOMAC	(3)–				

AT, acupuncture; AEs, adverse events; CCT, controlled clinical trials; HM, herbal medicine; IA, intra-articular injection; LKSS, Lysholm score; KOA, knee osteoarthritis; n.r., not reported; RCT, randomized controlled trials; ROB, risk of bias; VAS, visual analog scale; WA, warm acupuncture; western medicine; WOMAC, Western Ontario and McMaster Universities Osteoarthritis index.

^*^Total effective rate = (number of markedly effective cases + number of effective cases)/total cases.

^*^Relied on the original author’s judgment, (+): overall positive; (−): negative; (+/−): unclear.

### Overlap of reviews

A total of 15 SRs (12, 25–38) were included in this review. N indicates 245, r indicates 157, and c indicates 15. The formula CCA = (245–157)/(15× 157–157) = 0.04 indicates slight overlap. The overlap matrix is shown in Supplementary 3.

### Outcomes

Fifteen SRs (12, 25–38) summarized the evidence on the effectiveness of WA alone or in combination with Western medicine or traditional medicine in reducing pain and improving the total effective rate, function, and WOMAC total score. The outcomes from the included SRs are summarized and presented in Table 2.

**Table 2:** Certainty of evidence in included systematic review with GRADE approach.

**First author (year) [ref]**	**Outcomes**	**Study design**	**Number of studies (Total sample size)**	**Effect (95% CI)**	***p*-value**	**Certainty of evidence**
Feng (2019) (25)	Total effective rate	WA vs. WM	8 (582)	OR 4.10 [2.51, 6.71]	*< 0.00001*	Low
Guo (2018) (26)	Total treatment effect	WA vs. WM	11 (925)	OR 4.54 [3.02, 6.82]	*< 0.00001*	Low
	WOMAC total	WA vs. WM	2 (150)	SMD -0.68 [-1.02, -0.35]	*< 0.00001*	Very low
Lu (2015) (27)	Total effective rate	WA vs. WM	8 (811)	RR 1.37 [1.27, 1.48]	*< 0.0001*	Very low
	Total effective rate (long term)	WA vs. WM	2 (175)	RR 1.16 [1.04, 1.29]	*= 0.008*	Very low
	Total effective rate (short term)	WA vs. WM	4 (339)	RR 2.31 [1.57, 3.41]	*< 0.00001*	Low
	Adverse events	WA vs. WM	2 (176)	RR 0.20 [0.05, 0.75]	*= 0.02*	Low
Kong (2019) (28)	Total treatment effect	WA+WM vs. WM	16 (1445)	OR 4.20 [2.80, 6.32]	*< 0.00001*	Moderate
	Pain (VAS)	WA+WM vs. WM	11 (979)	MD -1.53 [-1.96, -1.11]	*< 0.00001*	Very low
	LKSS	WA+WM vs. WM	8 (753)	MD 19.7 [13.76, 25.18]	*< 0.00001*	Very low
	WOMAC total	WA+WM vs. WM	3 (312)	MD -10.25 [-15.61, -4.90]	*0.0002*	Very low
Cao (2019) (29))	Total effective rate	WA+WM vs. WM	9 (786)	RR 1.16 [1.10, 1.22]	*< 0.00001*	Moderate
Jun (2022) (12)	Total treatment effect	WA vs. WM (Drug)	24 (2278)	RR 1.22 [1.17, 1.27]	*< 0.001*	Low
		WA vs. WM (Injection)	5 (465)	RR 0.99 [0.91, 1.09]	NS	Low
		WA + WM (Drug) vs. WM (Drug)	8 (646)	RR 1.27 [1.18, 1.35]	*< 0.001*	Very Low
		WA + WM (Injection) vs. WM (Injection)	25 (2238)	RR 1.1.5 [1.11, 1.19]	*< 0.001*	Low
	Pain	WA vs. WM (Drug)	10 (874)	SMD -2.65 [-3.92, -1.38]	*= 0.01*	Very low
		WA vs. WM (Injection)	8 (726)	SMD -0.01 [-0.57, 0.55]	NS	Very low
		WA + WM (Drug) vs. WM (Drug)	2 (168)	SMD -5.85 [-7.84, -3.85]	*< 0.001*	Very low
		WA + WM (Injection) vs. WM (Injection)	19 (1795)	SMD -1.68[ -2.07, -1.29]	*< 0.001*	Very low
	Function	WA vs. WM (Drug)	13 (1354)	SMD -1.79 [-2.31, -1.26]	*< 0.001*	Very low
		WA vs. WM (Injection)	6 (547)	SMD -0.6 [-1.59, 0.39]	NS	Very low
		WA + WM (Drug) vs. WM (Drug)	4 (364)	SMD -1.45 [-3.11, 0.22]	*< 0.001*	Very low
		WA + WM (Injection) vs. WM (Injection)	22 (2012)	SMD -1.40 [-1.72, -1.08]	*< 0.001*	Very low
Jiang (2019) (30)	Total treatment rate	WA+WM vs. WM	20 (1719)	OR 4.45 [3.12, 6.35]	*< 0.00001*	Moderate
Chen (2019) (31)	Pain (VAS)	WA + other therapies vs. all type therapies	5 (450)	WMD -2.20 [-3.34, -1.06]	*< 0.05*	Very low
	WOMAC total	WA + other therapies vs. all type therapies	5 (418)	WMD -0.67 [-1.27, -0.07]	*< 0.05*	Very low
Huang (2021) (32)	Total effective rate	WA vs. all type therapies	17 (1109)	OR 3.41 [2.27, 5.13]	*< 0.00001*	Moderate
	Pain (VAS)	WA vs. all type therapies	10 (663)	MD -0.93 [-1.20, -0.67]	*< 0.00001*	Low
		WA vs. AT	4 (232)	MD -1.23 [-1.62, -0.84]	*< 0.00001*	Very low
		WA vs. EA	2 (130)	MD -0.63 [-1.09, -0.17]	*= 0.007*	Low
		WA vs. Moxa	3 (233)	MD -0.80 [-0.90, -0.70]	*< 0.00001*	Low
		WA vs. WM	1 (68)	MD -1.30 [-2.00, -0.60]	*= 0.0003*	Low
	WOMAC	WA vs. all type therapies	6 (430)	MD -8.91 [-12.58, -5.23]	*< 0.00001*	Low
		WA vs. AT	2 (172)	MD -14.11 [-20.89, -7.33]	*< 0.0001*	Very low
		WA vs. EA	2 (130)	MD -4.42 [-15.27, 6.44]	NS	Very low
		WA vs. WM	2 (128)	MD -8.29 [ -9.50, -7.08]	*< 0.0001*	Low
Luo (2019) (33)	Total treatment effect	WA vs. all type therapies	10 (819)	OR 5.22 [3.45, 7.89]	*< 0.00001*	Low
Jin (2022) (34)	Total effective rate	WA vs. TCM	8 (795)	RR 1.18 [1.06, 1.33]	*= 0.0004*	Low
	Daily activities	WA vs. TCM	2 (129)	MD -4.31 [-10.90, 2.28]	NS	Low
	Pain (VAS)	WA vs. TCM	6 (427)	MD -1.06 [-1.61, -0.51]	*= 0.0002*	Very low
	WOMAC	WA vs. TCM	6 (427)	MD -6.93 [ -12.14, -1.72]	*= 0.009*	Low
Li (2021) (35)	Total effective rate	WA vs. AT	10 (872)	OR 3.44 [2.25, 5.27]	*< 0.00001*	Moderate
		WA vs. EA	8 (643)	OR 0.91 [0.58, 1.43]	NS	Low
	Pain (VAS)	WA vs. AT	3 (178)	MD -1.40 [-1.83, 0.96]	*< 0.00001*	Low
		WA vs. EA	5 (374)	MD 0.82 [-0.08, 1.72]	NS	Very low
	WOMAC	WA vs. EA	4 (279)	MD 0.98 [-1.76, 3.71]	NS	Very low
	LKSS	WA vs. AT	2 (114)	MD 17.36 [13.40, 21.32]	*< 0.00001*	Low
Zhang (2018) (36)	Total effective rate	WA vs. TCM	12 (1176)	RR 1.04 [1.00, 1.08]	*= 0.06*	Very low
	Pain (VAS)	WA vs. TCM	7 (563)	SMD -0.48 [-1.26, 0.30]	NS	Very low
	LKSS	WA vs. AT	4 (359)	SMD -1.68 [-2.03, -1.32]	*< 0.001*	Low
	WOMAC total	WM vs. EA	4 (299)	SMD 0.69 [0.46, 0.92]	NS	Very low
Ou (2018) (37)	Total effective rate	WA vs. AT	7 (530)	OR 5.07 [2.85, 9.04]	*< 0.00001*	Low
Wu (2016) (38)	Total effective rate	WA vs. EA	11(772)	OR 1.21 [0.81, 1.80]	NS	Very low
	Pain (VAS)	WA vs. EA	5 (339)	SMD 0.25 [-0.11, 0.61]	NS	Very low
	WOMAC	WA vs. EA	4 (279)	SMD 0.08 [-0.15, 0.32]	NS	Very low

AT, acupuncture; GRADE, Grades of Recommendations, Assessment, Development, and Evaluation; LKSS, Lysholm Knee Score Scale; MD, mean difference; Moxa moxibustion; OR, odd ratio; ROB, risk of bias; RR, risk ratio; SMD, standard mean difference; TCM, traditional Chinese medicine; VAS, visual analog scale; WA, warm needle acupuncture.

#### Total effective rate

Fourteen SRs (12, 25–30, 32–38) suggested that the total effective rate of WA alone or combined with other therapies in OA patients was superior to that in the control group. One SR (30) with the largest sample size included 66 RCTs with 6,231 patients treatment, and a comparison of the effects of WA or WA plus WM group versus control group results showed a greater effect in the intervention group than in the control group. In most studies, WA was effective for OA. However, two SRs (35, 38) reported no significant differences between WA and EA.

#### Pain

Seven SRs (28, 31, 32, 34–36, 38) reported VAS scores. Four SRs (29, 31, 32, 34) had positive results, and three SRs (35, 36, 38) had negative results. Three SRs (28, 35, 36) reported the Lysholm score (LKSS) meta-analysis and showed that there was a significant difference between the WA alone or combined with other therapy groups and the control group. One SR (12) reported pain, which included the VAS, LKSS, and WOMAC (pain score). The SRs of the results were neither positive nor negative.

#### Function

One SR (12) evaluated the effects of WA alone or WA plus WM in the intervention group on function compared to WM. The analysis results of this SR showed that the intervention group was significantly improved compared with controls.

#### WOMAC total score

Seven SRs (28, 31, 32, 34–36, 38) reported the WOMAC total score. The meta-analysis showed the effects of WA alone or combined with other therapies on the WOMAC total score. However, four SRs (28, 31, 32, 34) failed to show that WA had superior effects compared with EA on the WOMAC.

#### Adverse events

Of all 15 SRs, five SRs (12, 27, 30, 33, 35) mentioned adverse events. The major symptoms reported in the WA treatment groups were skin burns. Most of the RCTs included in the SRs reported no adverse events. Four SRs (12, 30, 33, 35) reported that serious adverse events did not occur. One SR (27) indicated that the incidence of adverse events in the WA treatment groups was lower than that in the control groups, which indicated that WA was a safe therapy for OA.

### Methodological quality of the included systematic reviews

**Figure 2:**
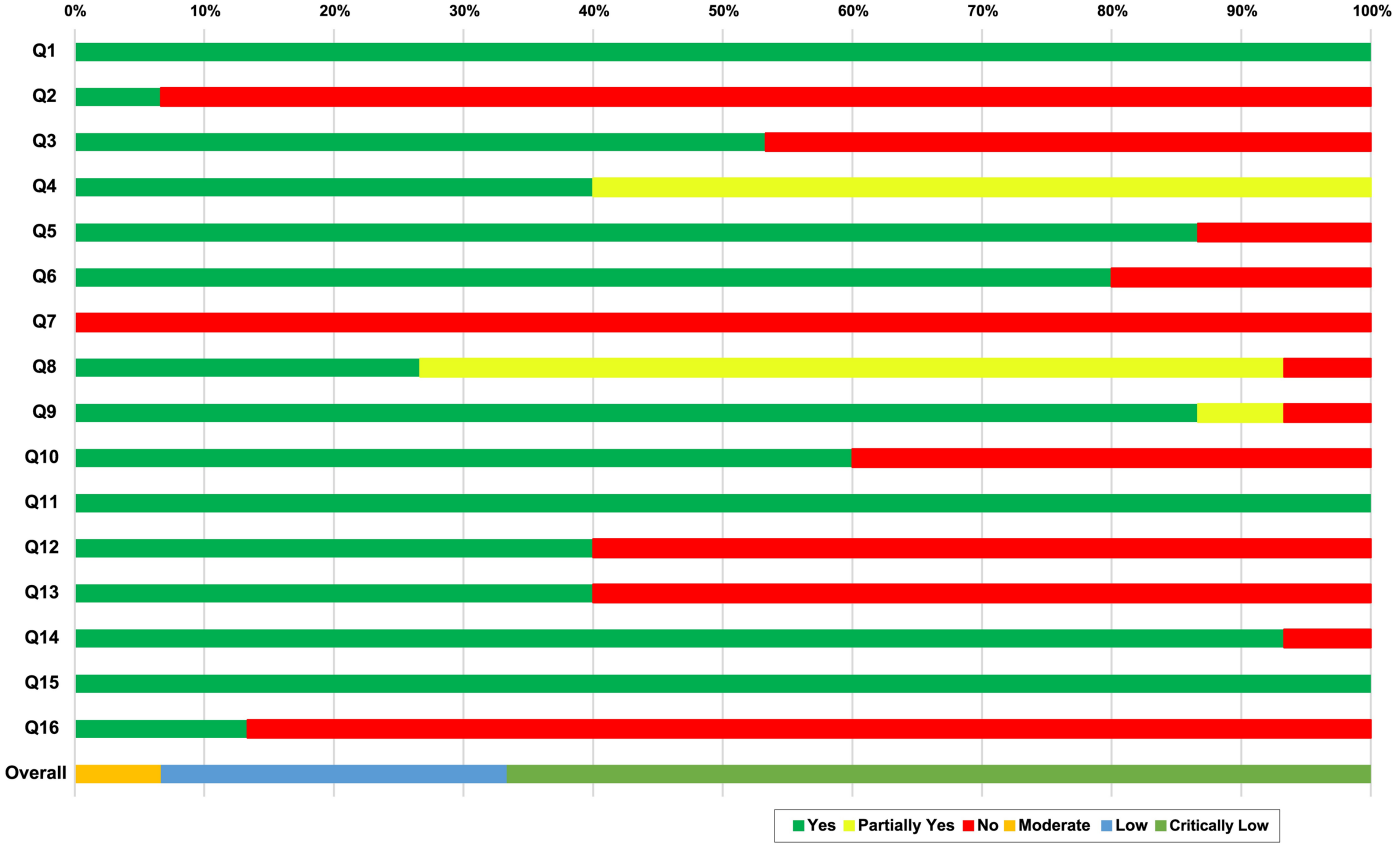
Quality evaluation using AMSTAR 2. AMSTAR2 was used to critically appraise the reporting quality of each included SR. The overall confidence of each SR was graded as “high” (no or non-critical weakness in all items), “moderate” (more than one non-critical weakness among all the items), “low” (one critical flaw with or without non-critical weakness), or “critically low” (more than one critical flaw with or without non-critical weakness). Q1: Did the research questions and inclusion criteria for the review included the components of PICO?; Q2: Did the report of the review contain an explicit statement that the review methods were established prior to the conduct of the review and did the report justify any significant deviations from the protocol?; Q3: Did the review authors explain their selection of the study designs for inclusion in the review?; Q4. Did the review authors use a comprehensive literature search strategy?; Q5: Did the review authors perform study selectin in duplicate?; Q6: Did the review authors perform data extraction in duplicate?; Q7: Did the review authors provide a list of excluded studies and justify the exclusions?; Q8: Did the review authors describe the included studies in adequate detail?; Q9: Did the review authors used a satisfactory technique for assessing the risk of bias (ROB) in individual studies that were included in the review?; Q10: Did the review authors report on the sources of funding for the studies included in the review?; Q11: If meta-analysis was performed did the review authors use appropriate methods for statistical combination of results? Q12: If meta-analysis was performed, did the review authors assess the potential impact of ROB in individual studies on the results of the meta-analysis or other evidence synthesis?; Q13: Did the review authors account for ROB in individual studies when interpreting/ discussing the results of the review?; Q14: Did the review authors provide a satisfactory explanation for, and discussion of, any heterogeneity observed in the results of the review?; Q15: If they performed quantitative synthesis did the review authors carry out an adequate investigation of publication bias (small study bias) and discuss its likely impact on the results of the review?; Q16: Did the review authors report any potential sources of conflict of interest, including any funding they received for conducting the review?. CL: critically low; L: low; M: moderate; PT: partial yes.

The results of the AMSTAR 2 tool showed that the included SRs were critically low quality, low quality, or moderate (Figure 2; Supplementary 4). Ten SRs (25–27, 29–31, 33, 36–38) were considered to have critically low quality, four SRs (28, 32, 34, 35) were considered to have low quality, and one SR (12) was considered to have moderate quality. All of the SRs reported the inclusion of PICO components (item 1). None of the SRs provided a complete list of excluded studies with reasons (item 7). Some SRs were evaluated with a partial yes in three domains (e.g., items 4 and 8).

Seven domains (items 2, 4, 7, 9, 11, 13, and 15) of the AMSTAR 2 tool were critical domains. For item 2, 14 of the SRs (25–38) provided a registry protocol, and one SR (12) was registered with PROSPERO and published protocol. For item 4, six SRs (12, 28, 30, 31, 34, 35) searched core databases (PubMed, the Cochrane Library, and Embase) and related intervention databases. However, nine SRs (25–27, 29, 32, 33, 36–38) lacked a search of the core databases. For item 7, none of the SRs provided the excluded studies and explained the reason for exclusion. For item 9, 13 SRs (25–30, 32–37) described the bias, one SR insufficiently reported bias (38), and one SR performed the assessment, but the results were not described. For item 11, all of the SRs performed a meta-analysis. For item 13, six SRs (12, 28, 33, 35–37) took the risk of bias into account when discussing the results and drew a conclusion with caution. For item 15, all of the SRs investigated publication bias and analyzed its potential effects on the results of the review.

### Report quality of included systematic reviews

**Figure 3:**
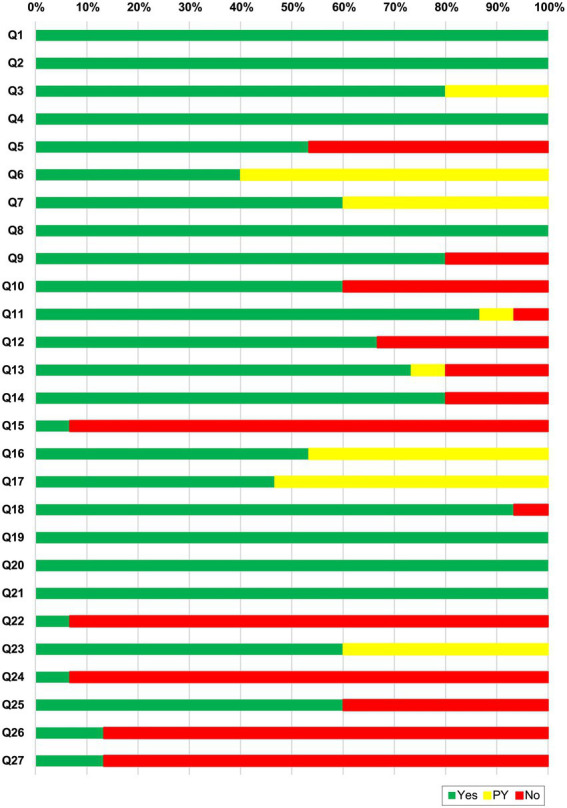
PRISMA 2020 checklist results for each item. Q1. Title; Q2. Abstract; Q3. Rationale; Q4. Objectives; Q5. Eligibility criteria; Q6. Information sources; Q7. Search strategy; Q8. Selection process; Q9. Data collection process; Q10. Data items; Q11. Study risk of bias assessment; Q12. Effect measures; Q13. Synthesis methods; Q14. Reporting bias assessment; Q15. Certainty assessment; Q16. Study selection; Q17. Study characteristics; Q18. Risk of bias in studies; Q19. Results of individual studies; Q20. Results of syntheses; Q21. Reporting biases; Q22. Certainty of evidence; Q23. Discussion; Q24. Registration and protocol; Q25. Support; Q27. Availability of data, code and other materials.

To assess the reporting quality of the included SRs, we used the PRISMA 2020 checklist (22). Figure 3 shows the reporting quality assessment results of the included SRs. Item 1 (title), item 2 (abstract), item 4 (objects), item 8 (selection process), item 19 (results of individual studies), item 20 (results of syntheses), and item 21 (reporting biases) were reported adequately (100%). Item 15 (describe any methods used to assess certainty (or confidence) in the body of evidence for an outcome), item 22 (certainty of evidence), and item 24 (registration and protocol) of results reported insufficient description. Overall, two SRs (12, 34) exhibited over 85% compliance. The results are shown in Supplementary 5.

### Certainty of evidence

We evaluated the quality of outcomes extracted from the included studies. Table 2 shows the level of evidence quality of the studies reported. The quality of evidence for outcomes evaluated by the GRADE approach ranged from very low to moderate (Supplementary 6). The risk of bias and imprecision mainly accounted for the downgrade. The quality of evidence was moderate for 5 outcomes (8.92%), low for 21 outcomes (35.21%), and very low for 32 outcomes (55.17%).

## Discussion

This overview of SRs was intended to summarize the features and evaluate the quality of methodological, reporting bias, and evidence from included SRs about the efficacy of WA in OA. Fifteen SRs reported that intervention groups using WA alone or WA plus other therapies showed symptom improvements compared with control groups (32, 35, 36, 38). WA treatment was safer than control treatment, and serious adverse events did not occur; however, the evidence of safety based on the included reviews was not sufficient since certain data were missing. Most of the SRs were associated with a high risk of bias, rated moderate to very low with the GRADE approach, and rated critically low with the AMSTAR 2 tool. Thus, it is not possible to draw a clear conclusion. Future research involving large sample sizes and high-quality studies are needed. Regarding the reporting quality of the results, only 2 SRs (12, 34) exhibited over 85% compliance.

All included studies had average reporting quality, according to the PRISMA 2020 checklist. The 6 element items (items 1, 2, 4, 8, 19, 20, and 21) were complete. Only two SRs reached 85.2% (34) and 100% ((12) compliance. Most of the included SRs were on knee OA and were conducted and published in China. In future studies, the reasonable utilization of the Consolidated Standards of Reporting Trials (CONSORT) (39) and PRISMA (22) checklists will improve the reporting quality of SRs and meta-analyses, which will reduce potential selection bias.

In nine SRs, the methodological quality was critically low because there were deficits in the critical items of the AMSTAR 2 tool, which included items 2 (registration protocol), 7 (list of excluded studies), and 16 (potential source of conflicts of interest). For item 2, only one SR (12) reported rates in the protocol and recording section. Preregistration helps to promote transparency, minimize potential biases in reporting and reviewing, reduce duplication of effort among groups, and keep service requests current. For item 7, an exclusion list is recommended because without this list, authors can arbitrarily exclude RCTs that differ from their desired results (21). Nevertheless, as the AMSTAR 2 tool is a more rigorous assessment tool than the previous version, the evaluation results should be interpreted by considering that the methodological quality of the published SRs was underestimated. A major reason for downgrading the evidence in the GRADE tool was that most of the included SRs were assessed as having a risk of bias and inconsistency across categories. The major reasons for this quality of evidence assessment were that randomization and blinding methods were not described and there was high heterogeneity.

This overview has some limitations. First, the SRs were dependent on RCTs published in China. The results of this review are not applicable or generalizable to other studies conducted elsewhere. In the future, clinical research should be actively conducted in countries other than China so that WA treatment for OA can be actively used in various ways. Second, the evaluation tools (AMSTAR, PRISMA, and GRADE) that were used were subjective. Two independent reviewers provided the evaluation, and the results were checked; nevertheless, they may have been their own judgment included in the assessment of each factor. Third, this overview was limited to the use of AMSTAR 2 to evaluate the methodological quality of the SRs. Consequently, the quality of the included SRs was not assessed. Future research should use the Risk of Bias in Systematic reviews (ROBIS) tool (40) to evaluate risk of bias and the PRISMA checklist (22) to evaluate the reporting characteristics of the included SRs.

In conclusion, WA or WA plus other therapies was more effective than the control conditions. However, the methodological quality of most of the included systematic reviews was critically low. Therefore, future studies should report SRs according to reporting guidelines, such as the PRISMA 2020 checklist, to improve the methodological quality and quality of evidence. This overview will help improve the evidence-based treatment and acupuncture evaluation system and facilitate research conducted by clinicians and scientific researchers.

## Data availability statement

The original contributions presented in the study are included in the article/Supplementary material, further inquiries can be directed to the corresponding author.

## Author contributions

JJ and ML: conceptualization, methodology, investigation, and writing—original draft. JJ: software, visualization, and project administration. T-YC and SP: validation and writing—review and editing. JJ and T-YC: formal analysis and resources. SP and ML: data curation and supervision. ML: funding acquisition. All authors read and approved the final manuscript.

## Funding

This research was supported by Korea Institute of Oriental Medicine (KSN 2022210). The authors alone are responsible for the writing and content of paper. The funder will not do any role for this study.

## Conflict of interest

The authors declare that the research was conducted in the absence of any commercial or financial relationships that could be construed as a potential conflict of interest.

## Publisher’s note

All claims expressed in this article are solely those of the authors and do not necessarily represent those of their affiliated organizations, or those of the publisher, the editors and the reviewers. Any product that may be evaluated in this article, or claim that may be made by its manufacturer, is not guaranteed or endorsed by the publisher.

## Footnotes

^1^ https://www.gradepro.org/
