# Supplementary material for: Warm needle acupuncture for osteoarthritis: An overview of systematic reviews and meta-analysis
Source: Front Med (Lausanne). 2023 Mar 14;10:971147. doi: 10.3389/fmed.2023.971147 (PMC10043310; doi:10.3389/fmed.2023.971147)
Supplement: Supplementary file 1 [file Data_Sheet_1.PDF]

Supplementary 1. Search strategies

1. PubMed (https://pubmed.ncbi.nlm.nih.gov/)

#1. “warm acupuncture” ti,ab

#2. “warm needle acupuncture” ti,ab

#3. “warm needling” ti,ab

#4. “warming needle moxibustion” ti,ab

#5. “warming acupuncture moxibustion” ti,ab

#6. “Warm acupuncture therapy” ti,ab

#7. “Needle warming therapy” ti,ab

#8. “Warm needle” ti,ab

#9. “Warming needle” ti,ab

#10. #1 OR #2 OR #3 OR #4 OR #5 OR #6 OR #7 OR #8 OR #9

#11. Osteoarthritis [MeSH Terms]

#12. osteoarthr* tw

#13. #11 OR #12

#14. “Meta-Analysis” pt OR “systematic review” pt

#15. “meta-analysis” ti,ab OR “systematic review” ti,ab

#16. #14 OR #15

#17. #10 AND #13 AND #16

2. Cochran library (https://www.cochranelibrary.com/)

#1. “warm acupuncture” ti,ab,kw

#2. “warm needle acupuncture” ti,ab,kw

#3. “warm needling” ti,ab,kw

#4. “warm acupuncture and moxibustion” ti,ab,kw

#5. “warming acupuncture and moxibustion” ti,ab,kw

#6. “warming needle moxibustion” ti,ab,kw

#7. “warming acupuncture moxibustion” ti,ab,kw

#8. “Warm acupuncture therapy” ti,ab,kw

#9. “Needle warming therapy” ti,ab,kw

#10. “Warm needle” ti,ab,kw

#11. “Warming needle” ti,ab,kw

#12. #1 OR #2 OR #3 OR #4 OR #5 OR #6 OR #7 OR #8 OR #9 OR #10 OR #11

#13. “Osteoarthritis”ti, ab, kw

#14. MeSH descriptor [osteoarthritis] explode all trees

#15. #13 OR #14

#16. (“Meta-Analysis”):pt OR (“systematic review”):pt OR “meta-analysis” ti,ab,kw OR “systematic review” ti,ab,kw

3. EMBASE (https://www.embase.com/)

S1: ti(“warm acupuncture”)

S2: ti(“warm needle acupuncture”)

S3: ti(“warm needling”) OR ab(“warm needling”)

S4: ti(“warm acupuncture and moxibustion”) OR ab(“warm acupuncture and moxibustion”)

S5: ti(“warming acupuncture and moxibustion”) OR ab(“warming acupuncture and moxibustion”)

S6: ti(“warming needle moxibustion”) OR ab(“warming needle moxibustion”)

S7: ti(“warming acupuncture moxibustion”) OR ab(“warming acupuncture moxibustion”)

S8: ti(“Needle warming therapy”) OR ab(“Needle warming therapy”)

S9: ti(“Warm needle”) OR ab(“Warm needle”)

S10: ti(“Warming needle”) OR ab(“Warming needle”)

S11: S10 OR S9 OR S8 OR S7 OR S6 OR S5 OR S4 OR S3 OR S2 OR S1

S12: ti(“Osteoarthritis”) OR ab(“Osteoarthritis”) OR emb(“Osteoarthritis”)

S13: ti(Meta-Analysis) OR ab(Meta-Analysis) OR pub(Meta-Analysis)

S14: ti(“systematic review”) OR ab(“systematic review”) OR pub(“systematic review”)

S15: S14 OR S13

S16 S15 AND S12 AND S11

4. CNKI (http://cnki.net)

#1. 温针 OR 温针灸 OR 温针疗法

#2. 骨关节炎 OR 骨性关节炎 OR 骨关节病 OR 关节炎

#3. 老年性关节炎 OR 老年性骨关节炎

#4. 退行性骨关节炎 OR 膝骨关节炎

#5. #2 OR #3 OR #4

#6. 系统评价 or meta

#7 #1 AND #5 AND #6

5. Wanfang (https: www.wanfangdata.com.cn/index.html)

#1. 温针 OR 温针灸 OR 温针疗法

#2. 骨关节炎 OR 骨性关节炎 OR 骨关节病 OR 关节炎

#3. 老年性关节炎 OR 老年性骨关节炎

#4. 退行性骨关节炎 OR 膝骨关节炎

#5. #2 OR #3 OR #4

#6. 系统评价 or meta

#7 #1 AND #5 AND #6

6. VIP (https://qikan.cqvip.com/)

#1. 温针 OR 温针灸 OR 温针疗法

#2. 骨关节炎 OR 骨性关节炎 OR 骨关节病 OR 关节炎

#3. 老年性关节炎 OR 老年性骨关节炎

#4. 退行性骨关节炎 OR 膝骨关节炎

#5. #2 OR #3 OR #4

#6. 系统评价 or meta

#7 #1 AND #5 AND #6

7. KISS (https://kiss.kstudy.com)

온침 AND 골관절염 AND 체계적 문헌고찰

온침 AND 골관절염 AND Meta

8. KTKP (https://www.koreantk.com/ktkp2014/)

온침 AND 골관절염 AND 체계적 문헌고찰

온침 AND 골관절염 AND Meta

9. RISS (https://www.riss.kr)

온침 AND 골관절염 AND 체계적 문헌고찰

온침 AND 골관절염 AND Meta

10. OASIS (https://www.oasis.kiom.re.kr)

온침 AND 골관절염 AND 체계적 문헌고찰

온침 AND 골관절염 AND Meta

11. DBPIA (https://www.dbpia.co.kr)

온침 AND 골관절염 AND 체계적 문헌고찰

온침 AND 골관절염 AND Meta

12. KMBASE (https://kmbase.medric.or.kr)

온침 AND 골관절염 AND 체계적 문헌고찰

온침 AND 골관절염 AND Meta

Supplementary 2. Exclude articles

No	First author year (ref)	Reasons for exclusion
1	Chai 2009 (1)	Comparing two different types of acupuncture
2	Lu 2012 (2)	Comparing two different types of acupuncture
3	Hu 2016 (3)	Comparing two different types of acupuncture
4	Zhang 2017 (4)	Comparing two different types of acupuncture
5	Wang 2019 (5)	Comparing two different types of acupuncture
6	Fan 2020 (6)	Comparing two different types of acupuncture
7	Sun 2020 (7)	Comparing two different types of acupuncture
8	Wen 2022 (8)	Comparing two different types of acupuncture
9	Zhao 2010 (9)	Data is missing
10	Ho 2018 (10)	Data is missing
11	Jun 2022 (11)	Data is missing
12	Zhang 2019 (12)	Wrong treatment
13	Yin 2021 (13)	Wrong treatment
14	Li 2022 (14)	Wrong treatment

Reference

1. Chai H, Li.B., Du YH. Meta-analysis for acupuncture and moxibustion treatment of knee osteoarthritis. Liaoning J Tridit Chin Med. 2009;36(7):1197-200.
2. Lu M, Tan XY, Huang L. Meta-analysis of acupuncture and moxibustion treatment for knee osteoarthritis. Guid J Trdit Chin Med Pharm. 2012;18(1):81-3.
3. Hu HJ. Meta-analysis of acupuncture and moxibustion treatment for knee osteoarthritis. Today Nurse. 2016(9):115-7.
4. Zhang YF. Meta-analysis of clinical effect of acupuncture on knee osteoarthritis [Master]. Liaoning: Liaoning Univ Tradit Chin Med; 2017.
5. Wang Y, Wang HF. Meta-analysis of clinical effect of acupuncture on knee osteoarthritis. Health Guide. 2019(12):9,11.
6. Fan MM, Fan YM, Li F, Liu Y, Guo JY. Meta-analysis for the efficacy and safety of acupuncture and moxibustion in the treatment of knee osteoarthritis. Rheumatism and Arthritis. 2020;9(5):26-31,40.
7. Sun N, Zou X, Lin LL, Shi GX, Wang LQ, Zhang T, et al. Systematic evaluation on acupuncture and mocibustion for treatment of knee osteoarthritis. Chin J Infor TCM. 2020;27(8):112-7.
8. Wen SB, Han J, Xu ZW. Meta-analysis of reticular pattern in the treatment of knee osteoarthritis with acupuncture and moxibustion. J Liaoning Univ TCM 2022;24(8):127-33.
9. Zhao Q, Feng W, Cai BJ. Systematic evaluation of therapeutic effect of warming acupuncture on osteoarthritis of knee joint in china. J Liaoning Univ TCM. 2010(4):49-51.
10. Ho SY. Meta analysis and systematic review for comparing the curative effect of warm needling with other traditional chinese medicine external medication on knee osteoarthrits. Guangzhou: Guangzhou Univ Chin Med; 2018.
11. Jun JH. Warm needle acupuncture for osteoarthritis: a systematic review of randomized controlled trials. Daejeon: Daejon Univ; 2022.
12. Zhang JR, Wang ZG, Yin XL. Meta analysis of warming acupuncture combined with intra-articular injection of sodium hyaluronate in treatment of knee osteoarthritis. J Shanxi Univ TCM. 2019;43(3):250-6.
13. Yin HH, Guo YH, Liu SQ, Liao ZJ, Xiao GT, Tang XD. Meta-analysis on the therapeutic effect of warm acupuncture and moxibution combined with traditional chinese medicine fumigation and washing on knee osteoarthrits. CJGMCM. 2021;36(7):1057-61.
14. Li J, Yang H, Hu T. Comparison of warming needle moxibustion and drug therapy for treating knee osteoarthritis: a systematic review and meta-analysis. Comput Math Methods Med. 2022;2022:3056109.

Supplementary 3. Overlap matrix

No	Randomized controlled trials	Systematic reviews														
	First author (year) (ref)	Feng 2019) (1)	Guo (2018) (2)	Lu (2015) (3)	Kong (2019) (4)	Cao (2019) (5)	Jun (2022) (6)	Jiang (2019) (7)	Chen (2019) (8)	Huang (2021)(9)	Luo (2019) (10)	Jin (2022) (11)	Li (2021) (12)	Zhang (2018) (13)	Ou (2018) (14)	Wu (2016) (15)
1	Bao (2019) (16)	0	0	0	0	0	0	0	0	1	0	0	0	0	0	0
2	Cao (2017) (17)	0	0	0	0	0	0	0	0	1	0	0	0	0	0	0
3	Chai (2015) (18)	0	0	0	0	0	0	0	0	0	1	0	0	0	0	0
4	Chang (2016) (19)	0	0	0	0	0	0	0	1	0	0	0	0	0	0	0
5	Chao (2018) (20)	0	0	0	0	0	1	0	0	0	0	0	0	0	0	0
6	Chen (2010) (21)	0	0	0	0	0	0	0	1	1	0	0	0	0	0	0
7	Chen (2012) (22)	0	0	0	0	0	0	0	1	0	0	0	0	0	0	0
8	Chen (2013) (23)	0	0	1	0	0	0	0	0	0	0	0	0	0	0	0
9	Chen (2015) (24)	0	0	0	0	0	0	1	0	0	0	0	0	0	0	0
10	Chen (2016) (25)	0	0	0	0	0	0	0	0	1	0	0	0	0	0	0
11	Chen (2017) (26)	0	0	0	1	0	1	1	0	0	0	0	0	0	0	0
12	Chen (2018) (27)	0	0	0	0	0	1	0	0	0	0	0	0	0	0	0
13	Chen (2018) (28)	0	0	0	0	0	1	0	0	0	0	0	0	0	0	0
14	Cui (2013) (29)	0	0	0	0	0	0	0	0	0	1	0	0	0	1	0
15	Dai (2018) (30)	0	0	0	0	0	0	1	0	0	0	0	0	0	0	0
16	Dang (2019) (31)	0	0	0	0	0	1	0	0	0	0	0	0	0	0	0
17	Ding (2009) (32)	1	1	0	0	0	1	0	0	0	0	0	0	0	0	0
18	Duan (2015) (33)	0	0	0	1	0	0	0	0	0	0	0	0	0	0	0
19	Fan (2018) (34)	0	0	0	0	0	0	0	0	1	0	0	0	0	0	0
20	Fei (2005) (35)	0	1	0	0	0	0	0	0	0	0	0	0	0	0	0
21	Gao (2012) (36)	0	0	0	0	0	0	0	0	0	0	1	1	1	0	1
22	Gao (2017) (37)	0	0	0	1	1	1	1	0	0	0	0	0	0	0	0
23	Guo (2013) (38)	0	0	0	0	0	0	0	1	0	0	0	0	0	0	0
24	Guo (2019) (39)	0	0	0	0	0	1	0	0	0	0	0	0	0	0	0
25	Han (2013) (40)	0	0	1	0	0	0	0	0	0	0	0	0	0	0	0
26	Han (2016) (41)	0	0	0	1	0	1	1	0	0	0	0	0	0	0	0
27	Han (2018) (42)	0	0	0	0	0	0	0	0	1	0	1	0	0	0	0
28	Han (2019) (43)	0	0	0	0	0	1	0	0	0	0	0	0	0	0	0
29	Han (2021) (44)	0	0	0	0	0	1	0	0	0	0	0	0	0	0	0
30	He (2017) (45)	0	0	0	0	0	0	0	1	0	0	0	0	0	0	0
31	He (2018) (46)	0	0	0	1	0	1	0	0	0	0	0	0	0	0	0
32	Hou (2020) (47)	0	0	0	0	0	1	0	0	0	0	0	0	0	0	0
33	Hu (2015) (48)	0	0	0	0	0	0	0	0	0	0	0	1	0	0	0
34	Hu (2015) (49)	0	0	0	0	0	0	0	0	1	0	0	0	0	0	0
35	Hu (2016) (50)	0	0	0	0	0	1	0	0	0	0	0	0	0	0	0
36	Hui (2015) (51)	0	0	0	0	0	0	0	1	0	0	0	0	0	0	0
37	Ji (2016) (52)	0	0	0	0	0	1	0	0	0	0	0	0	0	0	0
38	Jiang (2004) (53)	0	0	0	0	0	0	0	0	0	0	0	0	0	0	1
39	Jiang (2013) (54)	0	1	0	0	0	0	0	0	0	0	0	0	0	0	0
40	Jiang (2014) (55)	0	0	0	0	1	1	1	0	0	0	0	0	0	0	0
41	Jiang (2016) (56)	0	0	0	0	0	0	0	0	0	1	0	1	0	1	0
42	Jin (2016) (57)	0	0	0	0	0	0	0	0	1	0	0	0	0	0	0
43	Kong (2017) (58)	0	0	0	1	0	0	0	0	0	0	0	0	0	0	0
44	Kuang (2001) (59)	0	0	0	0	0	0	0	0	0	1	0	0	0	0	0
45	Li (2006) (60)	0	0	0	0	0	0	0	1	0	0	1	1	1	0	0
46	Li (2010) (61)	0	0	0	0	0	0	0	0	1	0	0	0	0	0	0
47	Li (2010) (62)	0	0	0	0	0	0	0	0	0	1	0	0	0	0	0
48	Li (2015) (63)	1	0	0	0	0	0	0	0	0	0	0	0	0	0	0
49	Li (2017) (64)	0	1	0	0	0	0	0	0	0	0	0	0	0	0	0
50	Li (2018) (65)	0	0	0	1	1	1	1	0	0	0	0	0	0	0	0
51	Liang (2012) (66)	0	0	0	1	1	1	1	0	0	0	0	0	0	0	0
52	Liang (2016) (67)	0	0	0	1	0	1	1	0	0	0	0	0	0	0	0
53	Liang (2018) (68)	0	0	0	0	0	1	1	0	0	0	0	0	0	0	0

**54**	Lin (2019) (69)	0	0	0	0	0	0	0	0	0	0	0	1	0	0	0
**55**	Lin (2020) (70)	0	0	0	0	0	1	0	0	0	0	0	0	0	0	0
**56**	Liu (2014) (71)	0	0	0	1	1	1	1	0	0	0	0	0	0	0	0
**57**	Liu (2015) (72)	0	0	0	0	0	0	0	0	0	0	0	0	1	0	0
**58**	Liu (2016) (73)	0	0	0	0	0	0	0	1	0	0	0	0	0	0	0
**59**	Liu (2016) (74)	0	1	0	0	0	0	0	0	0	0	0	0	0	0	0
**60**	Liu (2017) (75)	1	0	0	0	0	0	0	0	0	0	0	0	0	0	0
**61**	Liu (2017) (76)	1	0	0	0	0	0	0	0	0	0	0	0	0	0	0
**62**	Liu (2018) (77)	0	0	0	0	0	0	0	0	0	0	0	0	0	0	0
**63**	Lu (2014) (78)	0	0	0	0	0	0	0	0	0	0	0	1	1	0	1
**64**	Ma (2013) (79)	0	0	0	0	0	1	0	0	0	0	0	0	0	0	0
**65**	Ma (2015)(80)	0	0	0	0	0	0	0	1	0	0	0	0	0	1	0
**66**	Ma (2016) (81)	0	0	0	0	0	1	0	0	0	0	0	0	0	0	0
**67**	Ma (2020) (82)	0	0	0	0	0	1	0	0	0	0	0	0	0	0	0
**68**	Ma (2021) (83)	0	0	0	0	0	1	0	0	0	0	0	0	0	0	0
**69**	Mei (2014) (84)	0	0	0	0	0	0	0	0	0	1	0	0	0	0	0
**70**	Mo (2015) (85)	0	0	0	0	0	0	0	0	0	0	1	0	0	1	0
**71**	Niu (2014) (86)	0	0	0	0	0	0	0	0	0	0	0	0	0	0	1
**72**	Ou (2011) (87)	0	0	0	0	0	0	0	0	1	0	0	1	1	0	1
**73**	Ou (2018) (88)	0	0	0	0	0	1	1	0	0	0	0	0	0	0	0
**74**	Pan (2014) (89)	0	0	0	0	0	0	0	0	1	0	1	0	0	0	0
**75**	Pan (2020) (90)	0	0	0	0	0	0	0	0	0	0	1	0	0	0	0
**76**	Peng (2009) (91)	0	0	0	0	0	0	0	0	0	0	0	1	0	0	0
**77**	Qi (2017) (92)	0	0	0	0	0	0	0	0	1	0	0	0	0	0	0
**78**	Qiao (2018) (93)	0	0	0	0	0	1	0	0	0	0	0	0	0	0	0
**79**	Qin (2013) (94)	1	0	0	0	0	1	0	0	0	0	0	0	0	0	0
**80**	Qiu (2013) (95)	0	1	0	0	0	1	0	0	0	0	0	0	0	0	0
**81**	Qiu (2013) (96)	0	0	0	0	0	0	0	0	0	0	0	1	0	0	1
**82**	Qu (2015) (97)	0	0	0	0	0	0	0	0	0	0	1	1	1	0	0
**83**	Ren (2012) (98)	0	0	0	1	1	1	1	0	0	0	0	0	0	0	0
**84**	Shi (2016) (99)	0	0	0	1	0	1	0	0	0	0	0	0	0	0	0
**85**	Shu (2014) (100)	0	0	0	1	0		0	0	0	0	0	0	0	0	0
**86**	Shu (2021) (101)	0	0	0	0	0	1	0	0	0	0	0	0	0	0	0
**87**	Si (2013) (102)	0	0	0	1	0	0	0	0	0	0	0	0	0	0	0
**88**	Song (2016) (103)	0	0	0	0	0	1	0	0	0	0	0	0	0	0	0
**89**	Su (2020) (104)	0	0	0	0	0	1	0	0	0	0	0	0	0	0	0
**90**	Sun (2012) (105)	0	0	0	0	0	0	0	0	0	0	0	0	1	0	0
**91**	Teng (2012) (106)	0	0	0	0	0	0	0	0	0	0	0	0	0	0	1
**92**	Teng (2020)(107)	0	0	0	0	0	1	0	0	0	0	0	0	0	0	0
**93**	Tian (2006)(108)	0	0	1	0	0	0	0	0	0	0	0	0	0	0	0
**94**	Tu (2016) (109)	0	0	0	0	0	1	0	0	0	1	0	0	0	0	0
**95**	Wang (2007) (110)	0	0	0	0	0	0	0	1	0	0	0	0	1	0	0
**96**	Wang (2010) (111)	0	0	0	1	0	1	1	0	0	0	0	0	0	0	0
**97**	Wang (2011) (112)	0	0	0	0	0	0	1	0	0	0	0	0	0	0	0
**98**	Wang (2015) (113)	0	0	0	0	0	0	0	1	0	0	0	1	1	0	0
**99**	Wang (2015) (114)	0	0	0	0	0	0	0	0	1	0	0	0	0	0	0
**100**	Wang (2015) (115)	0	0	0	1	0	0	0	0	0	0	0	0	0	0	0
**101**	Wang (2016) (116)	0	1	0	0	0	1	0	0	0	0	0	0	0	0	0
**102**	Wang (2017) (117)	0	0	0	0	0	0	0	0	1	0	0	1	0	0	0
**103**	Wang (2017) (118)	0	0	0	0	0	0	0	0	0	0	0	1	0	0	0
**104**	Wang (2017) (119)	0	0	0	0	0	0	0	1	0	1	0	0	0	0	0
**105**	Wang (2018) (120)	0	0	0	0	0	1	0	0	0	0	0	0	0	0	0
**106**	Wang (2019) (121)	0	0	0	0	0	1	0	0	0	0	0	0	0	0	0
**107**	Wang (2019) (122)	0	0	0	0	0	1	0	0	0	0	0	0	0	0	0
**108**	Wei (2013) (123)	0	0	0	0	0	1	0	0	0	0	0	0	0	0	0
**109**	Wu (2006) (124)	0	1	1	0	0	1	0	0	0	1	0	0	0	0	0
**110**	Wu (2009) (125)	0	0	0	0	0		0	0	0	0	1	0	0	0	0
**111**	Wu (2013) (126)	0	0	0	0	0	1	0	0	0	0	0	0	0	0	0
**112**	Wu (2013) (127)	0	0	0	0	0	0	0	1	1	0	0	0	0	0	0
**113**	Wu (2016) (128)	0	0	0	0	0	0	0	0	0	0	0	0	0	1	0

**114**	Wu (2017) (129)	0	0	0	1	1	0	1	0	0	0	0	0	0	0	0
**115**	Wu (2018) (130)	0	0	0	0	0	1	0	0	0	0	0	0	0	0	0
**116**	Xia (2012) (131)	0	0	0	0	0	0	0	0	0	0	0	0	0	0	1
**117**	Xu (2014) (132)	1	0	0	0	0	0	0	0	0	0	0	0	0	0	0
**118**	Xu (2019) (133)	0	0	0	0	0	1	0	0	0	0	0	0	0	0	0
**119**	Xu (2020) (134)	0	0	0	0	0	1	0	0	0	0	0	0	0	0	0
**120**	Xue (2011) (135)	0	1	1	0	0	1	0	0	0	0	0	0	0	0	0
**121**	Xue (2015) (136)	0	0	0	0	0	1	0	0	0	0	0	0	0	0	0
**122**	Yang (2012) (137)	0	0	1	0	0	1	0	0	0	0	0	0	0	0	0
**123**	Yang (2014) (138)	0	0	0	0	0	0	0	1	1	0	0	0	0	0	0
**124**	Yang (2015) (139)	0	0	0	0	0	0	0	1	0	0	0	0	0	0	0
**125**	Yang (2015) (140)	0	0	0	0	0	0	0	0	0	0	0	1	0	0	0
**126**	Yao (2018) (141)	0	0	0	0	0	1	0	0	0	0	0	0	0	0	0
**127**	Ying (2015) (142)	0	0	0	0	0	0	0	0	0	0	0	1	0	0	0
**128**	Yu (2009) (143)	0	0	0	0	0	0	0	0	0	0	0	0	0	1	0
**129**	Yu (2016) (144)	0	0	0	0	0	1	0	0	0	0	0	0	0	0	0
**130**	Yu (2016) (145)	0	0	0	1	1	1	1	0	0	0	0	0	0	0	0
**131**	Zeng (2016) (146)	0	0	0	0	0	0		1	0	0	0	0	0	1	0
**132**	Zeng (2017) (147)	0	0	0	1	0	0	1	0	0	0	0	0	0	0	0
**133**	Zhang (2009) (148)	0	0	0	0	0	0	0	0	0	0	0	0	1	0	0
**134**	Zhang (2011) (149)	0	0	0	1	1	1	0	0	0	0	0	0	0	0	0
**135**	Zhang (2013) (150)	0	0	0	0	0	0	0	0	0	0	0	0	0	0	1
**136**	Zhang (2013) (151)	0	0	0	0	0	0	0	1	0	0	0	0	0	0	0
**137**	Zhang (2013) (152)	0	0	0	0	0	0	0	0	0	0	0	1	1	0	1
**138**	Zhang (2014) (153)	0	0	0	0	0	1	0	0	0	0	0	0	0	0	0
**139**	Zhang (2016) (154)	0	0	0	0	0	0	0	1	0	0	0	0	0	0	0
**140**	Zhang (2016) (155)	0	0	0	0	0	0	0	0	0	1	0	0	0	0	0
**141**	Zhang (2016) (156)	0	0	0	0	0	1	0	0	0	0	0	0	0	0	0
**142**	Zhang (2017) (157)	1	0	0	0	0	1	0	0	0	0	0	0	0	0	0
**143**	Zhang (2018) (158)	0	0	0	0	0	1	1	0	0	0	0	0	0	0	0
**144**	Zhang (2019) (159)	0	0	0	0	0	1	0	0	0	0	0	0	0	0	0
**145**	Zhang (2019) (160)	0	0	0	0	0	1	0	0	0	0	0	0	0	0	0
**146**	Zheng (2008) (161)	0	0	0	0	0	1	0	0	0	0	0	0	0	0	0
**147**	Zheng (2016)(162)	0	0	0	0	0	1	0	0	0	0	0	0	0	0	0
**138**	Zhong (2008) (163)	0	0	0	0	0	0	0	0	0	0	0	0	0	0	0
**149**	Zhou (2006)(164)	0	1	1	0	0	0	0	0	0	0	0	0	0	0	0
**150**	Zhou (2014) (165)	0	0	0	0	0	0	0	0	0	0	0	1	1	0	1
**151**	Zhou (2014) (166)	0	0	0	0	0	0	0	0	1	0	0	0	0	0	0
**152**	Zhou (2015) (167)	0	1	0	0	0	0	0	0	0	0	0	0	0	0	0
**153**	Zhou (2020) (168)	0	0	0	0	0	1	0	0	0	0	0	0	0	0	0
**154**	Zhu (2011) (169)	0	0	1	0	0	0	0	0	0	0	0	0	0	0	0
**155**	Zhu (2017) (170)	1	0	0	0	0	0	0	0	0	0	0	0	0	0	0
**156**	Zuo (2011) (171)	0	0	0	0	0	1	0	0	0	0	0	0	0	0	0
**157**	Zuo (2015) (172)	0	0	0	1	0	1	1	0	0	0	0	0	0	0	0

Reference

1. Feng P, Gao YL, Yang J, Xu J, Su YL, Yu JJ, et al. Meta analysis of clinical efficacy of acupuncture and moxibustion in the treatment of knee osteoarthritis. Sci Tech Information Gansu 2019;48(3):87-9.
2. Guo KY, Chen DL. Efficacy of needle warming therapy for treatment of knee osteoarthritis: a systematic review. J Tradit Chin Orthop Traumatology. 2018;30(7):17-20.
3. Lu W. Needle warming moxibustion versus oral western medicine for knee osteoarthritis：a systematic review and meta-analysis. J Trauma Emergency (Electronic Ver). 2015(2):30-4.
4. Kong YY, Jing MY, Qin Y, Chen PJ, Ren KJ. Effect of warm acupuncture combined with sodium hyaluronate injection on knee osteoarthrits. World Latest Med Information. 2019;19(20):27-32,5.
5. Cao J, Zhou Q, Qian QR. Clinical curative effect of combianton therpy of acupuncture and intra-articular injection of sodium hyaluronate versus monotherapy of intra-articular injection of sodium hyaluronate for knee osteoarthritis: a systematic review. J tradit Chin Orthop Trauma. 2019;31(3):33-7.
6. Jun JH, Choi TY, Robinson N, Park JY, Jun EY, Kim KH, et al. Warm needle acupuncture for osteoarthritis: A systematic review and meta-analysis. Phytomed. 2022;106.
7. Jiang H, Zhang S. Meta-analysis of warm acupuncture combined with sodium hyaluronate therapy in treatment of knee osteoarthritis. Chin Med Herald. 2019;16(6):154-8.
8. Chen YL, Zhou QL, Ji SL, Sun WP, Chen JT. Efficacy and safety of warming needle moxibustion in treating knee osteoarthritis: a meta -analysis. Chin J Ethnomedicine Ethnopharmacy. 2019;28(2):45-50.
9. Huang ZL, Shi SN, He JJ, Gao HY, Hong ZQ. Meta-analysis of trherpeutic effect of warm acupuncture and moxibustion on knee osteoarthritis of ynag deficiency and cold coagulation and research on regularity of acupoint selection. Rehabilit Med. 2021;31(4):341-50.
10. Luo M, Tan J, Zhu YL, Sun L, Lan W, Zhu ZZ, et al. Meta analysis of therapeutic effect of warm acupuncture and moxibustion on knee osteoarthritis. Hunan J Tradit Chin Med. 2019;35(3):120-3.
11. Jin S, Guan X. A systematic review and meta-analysis of the comparative curative effects of warm acupuncture and other traditional Chinese medicines in the treatment of knee osteoarthritis. Ann Palliat Med. 2022;11(2):708-16.
12. Li XJ, Lei MN, Jiang ZM, Zhao LN, Hu L, Wu ZJ. A meta analysis of warming needle acupuncture in the treatment of knee osteoarthrits. Henan TCM. 2021;41(2):250-60.
13. Zhang JW. Meta analysis of randomized controlled trials of warming needle moxibustion for knee osteoarthrits. Clin J TCM. 2018;30(11):2050-4.
14. Ou Y, J.J., Liu ZF, Wu Y, Cai WP, Gu X. Meta-analysis of clincial efficacy of warm acupuncture and moxibustion in the treatment of knee osteoarthrits. Hunan J TCM. 2018;34(4):128-9.
15. Wu Y, Xie ZY, Sun SQ, Li HN, Tan T. Meta-analysis of curative effect of needle warming moxibustion and electroacupuncture on knee osteoarthritis. Shandong J TCM 2016;35(4):320-4.
16. Bao GX. Clinical study on warm acupuncture treatment of knee osteoarthritis of yang deficiency cold condensate type. World Latest Med Inf. 2019;19(77):180-1.
17. Cao S. Warm acupuncture treatment of Yang deficiency type haemorrheological nature of knee osteoarthritis clinical studies. Changchun: Changchun Univ Chin Med; 2017.
18. Chai GH. Observation on therapeutic effect of warming acupuncture on knee osteoarthritis. Chin Man-Rehabil Med. 2015;6(23):51-2.
19. Chang JJ, Jiang T, Yang JZ, Yu GY, Li Y. 40 cases of knee osteoarthritis of cold and damp type treated by warm acupuncture and moxibustion. Yunnan J Tradit Chin Med Materia Med. 2016;37(3):50-1.
20. Chao JG. Clinical randomized controlled study on warm acupuncture and moxibustion in the treatment of knee osteoarthritis Diabet World. 2018;15(9):40.
21. Chen SM. The clincial research on knee osteoarthritis treated by warm needle moxibustion. Guangzhou: Guangzhou Univ Chin Med; 2010.
22. Chen ZG, Wu LH, Chen MJ, Wang C. Therapeutic Observation on Warm-needling Therapy for Knee Osteoarthritis. Shanghai J Acu-mox. 2012;31(5):339-41.
23. Chen TY. Clinical observation on the treatment of knee osteoarthritis with warm acupuncture. Healthways. 2013;12(8):433.
24. Chen JD. Clinical observation on 100 cases of knee osteoarthritis treated with sodium hyaluronate combined with acupuncture. Women's Health Research. 2015(7):197,210.
25. Chen J. The clinical research on treatment of knee osteoarthritis of the Yang deficiency and cold coagulation type by warm needle moxibustion. Hefei: Anhui Univ Chin Med; 2016.
26. Chen J, Shen J, Chen Z, Hu W. Efficacy of warming acupuncture combined with sodium hyaluronate intraarticular injection in the treatment of osteoarthritis and ithe influence on MMPs. World Chin Med. 2017;12(12):3102-5.
27. Chen J, Wang J, Zhao K, Zhang GC. Clinical observation of warming acupuncture and moxibustion on knee osteoarthritis of Yang deficiency and cold coagulation type J Shanxi Univ Chin Med. 2018;41(6):88-90,6.
28. Chen F, Zhu Y, Shi X. Theraputic effect of warm acupuncture on knee osteoarthritis at different stages. Shanxi J Tradit Chin Med. 2018;39(7):953-5.
29. Cui HW. Analysis of therapeutic effect of warming acupuncture on knee osteoarthritis. Guide Chin Med. 2013;11(14):669-70.
30. Dai LL, Hou ZZ. Clinical observation on 30 cases of knee osteoarthritis treated with warm acupuncture and sodium hyaluronate. Zhejiang J Tradit Chin Med. 2018;53(03):209.
31. Dang CQ. Observation on the effect of acupuncture and moxibuastion on knee osteoarthrits. Inner Mongolia JTradit Chin Med. 2019;38(12):125-6.
32. Ding MH, Zhang H, Li Y. A randomized controlled study on warming needle moxibustion for treatment of knee osteoarthritis. Chin Acu-Mox. 2009;29(8):603-7.
33. Duan S. Clinical observation on the therapeutic effect of warming acupuncture on knee osteoarthritis. Asia Pac Tradit Med. 2015;11(15):107-8.
34. Fan XH. Analgesic effect of different warm moxibustion methods on knee osteoarthritis of yang deficiency and cold congealing. Internat J Nurs. 2018;37(17):2432-4.
35. Fei M. 42 cases of knee degenerative osteoarthritis treated with warm acupuncture. J Clin Acu Mox. 2005(04):40-1.
36. Gao J, Ouyang BS, Zhang Y. Comparison of the clinical therapeutic effects between electroacupuncture and warming needle moxibustion for knee osteoarthritis of kidney deficiency and marrow insufficiency pattern/syndrome. Zhongguo Zhen Jiu. 2012;32:395-8.
37. Gao Y. Effect of intra-articular injection of sodium hyaluronate combined with acupuncture on knee osteoarthritis Chin J Convalecent Med. 2017;26(12):1258-60.
38. Guo Y. Clinical analysis of 200 cases of knee osteoarthritis treated with warm acupuncture. Guide Chin Med. 2013(32):504-5.
39. Guo M, Ding X. Clinical observation on warm acupuncture and moxibustion in the treatemtn of pain of knee osteoarthritis. CJGMCM. 2019;34(12):1873-4, 86.
40. Han YA. Observation on 28 cases of knee osteoarthritis treated with warm acupuncture and moxibustion. J Pract Tradit Chin Med. 2013;29(04):272-3.
41. Han D, Zhang H. Warm acupuncture combined with articular injection of sodium hyaluronate in treating knee osteoarthritis for 64 cases. Chin Med Mod Edu 2016;14(8):112-3.
42. Han YL, Chen S, Pan XL. The clinical research into knee osteoarthritis of Yang deficiency cold condensate type treated with warm acupuncture and moxibustion. Henan Tradit Chin Med. 2018;38(7):1096-9.
43. Han W. Clinical study on warm needling combined with intra-articular injection of sodium hyaluronate for knee osteoarthritis J New Chin Med. 2019;51(12):242-5.
44. Han NY. Analysis on the effecacy and safety of warm acupuncture and moxibustion in patients with knee osteoarthritis Chin Healthcare Nutr. 2021;31:210.
45. He XH, Zhang SM. Influence comparison of WOMAC score and pain symptoms of different acupuncture styles on cold type of knee osteoarthritis patients Shanxi J Tradit Chin Med. 2017;38(4):511-2.
46. He B, Chen X, Zhan X. Effect of warm acupuncture combined with intra-articular injection of sodium hyaluronate on knee function and VAS score in patients with knee osteoarthritis. Chin Med Innov. 2018;15(4):73-6.
47. Hou H, Shi S, Xue L, Shi Y. Influence of warming acupuncture and loxoprefen sodium on bone metablism, IL-6,LI1 and SOD in acute exacerbation of knee osteoarthritis. West J Tradit Chin Med. 2020;33(11):125-8.
48. Hu JH. Analysis of therapeutic effect of warm acupuncture on knee osteoarthritis of cold-damp type. J Clin Acu Mox. 2015(8):30-1.
49. Hu DH. Therapeutic effect of warm acupuncture and moxibustion on knee osteoarthritis of Yang deficiency and cold coagulation. Asia Pac Tradit Med. 2015;11(21):102-3.
50. Hu L, Tu X, Huang F, Yang E. Clinical reserch on the treatment of knee osteoarthritis with chinese and western medicine. J Pract Med Tech. 2016;23(4):347-9.
51. Hui W. Clinical analysis of 200 cases of knee osteoarthritis treated with warm acupuncture. World Latest Med Inf. 2015;15(97):150-1.
52. Ji WL. Clinical observation on 96 cases of knee osteoarthritis treated with warm acupuncture and moxibustion. J Erontiers Med. 2016;6(10):187-8.
53. Jiang ZH. Clinical observation of senium osteoarthritis treated with warm needle mainly. J Clin Acu Mox. 2004;20(7):40-1.
54. Jiang B, Hou XQ, Tang ZY. A Randomized Controlled Study on Warming Needle Moxibustion for the Treatment of Knee Osteoarthrit. Chin J Trad Med Traumatol-Orthop. 2013;21(11):18-21.
55. Jiang NM. Observation on therapeutic effect of acupuncture combined with sodium hyaluronate on knee osteoarthritis Guangxi J Tradit Chin Med. 2014;37(5):46-8.
56. Jiang H. Clinical observation on therapeutic effect of warm acupuncture and moxibustion on senile osteoarthritis of knee. Shanxi J Tradit Chin Med. 2016;37(1):99-100.
57. Jin.X.P. Comparative analysis of warm acupuncture and common acupuncture in the treatment of knee osteoarthritis caused by Yang deficiency and cold congealing. Health Care Guide. 2016;35:201.
58. Kong WL. Analysis of the effect of sodium hyaluronate injection combined with warm acupuncture on knee arthritis. Contemp Med Symp. 2017;15(18):93-4.
59. Kuang HJ, Huang TY, Wang FC. Observation on therapeutic effect of warming acupuncture on 52 cases of degenerative knee arthropathy. Massage Guided. 2001;17(4):40-1.
60. Li CD, Huang XY, Yang XG, Wang QF, Huang SQ. Observation on therapeutic effect of warming acupuncture on knee osteoarthritis of deficiency cold type. Chin Acu-Mox. 2006;26(3):189-91.
61. Li L, Hong KD, Wu MX. Clinical observation of knee osteroarthritis of tang deficiency and cold coagulation type treated by warm needle moxibuation. Fujian J Tradit Chin Med. 2010;41(5):36-7.
62. Li BH. Clinical research for knee osteoarthritis acupuncture and moxibustion treatment [Master]. Nanjing: Nanjing Univ Trad Chin Med; 2010.
63. Li ZM, Wu WC, Liu MY. Clinical observation on the treatment of knee osteoarthritis with warm acupuncture. World Latest Med Inf. 2015;15(14):143+7.
64. Li H. Observation on therapeutic effect of acupuncture and moxibustion on knee osteoarthritis. J Clin Med Literature. 2017;4(14):2617.
65. Li HT, Chao CZ, Song ML. Clinical observation of warm acupuncture combined with intra-articular injection of sodium hyaluronate in the treatment of knee osteoarthritis. Asia Pac Tradit Med. 2018;14(1):166-8.
66. Liang YP, Liang YF. Clinical observation on 30 cases of knee osteoarthritis treated with warm needling combined with intraarticular injection of Sodium Hyaluronate Injection. J New Chin Med. 2012;44(7):128-9.
67. Liang JL, Li L. Clinical study of sodium hyaluronate combined with warm acupuncture and moxibustion in the treatment of cold dampness obstruction type secondary KOA Mod Tradit Chin Med. 2016;36(2):33-5.
68. Liang TY. Acupuncture combined with sodium hyaluronate in the treatment of 50 cases of knee osteoarthritis TCM Res. 2018;31(5):55-7.
69. Lin WL, Chen YY. Clinical observation on the treatment of knee osteoarthritis with warm acupuncture. Chin J Trad Med Scie Technol. 2019;26(3):436-7.
70. Lin SL, Ye M. Efficacy of needle warming moxibustion combined with celecoxib on knee osteoarthritis and its effects on levels of seum MMP-3, TIMP-1, TMP-1, and IGF-1. Chin J Gen Pract. 2020;18(3):476-9.
71. Liu P, Wang Q, Chen Z, Li L. Observations on the efficacy of needle-sticking warm needling moxibustion plus sodium hyaluronate in treating knee osteoarthritis. Shanghai J Acu-

mox. 2014;33(12):1152-5.
72. Liu GP, Wang XH. Clinical comparison of warm acupuncture and moxibustion+electroacupuncture+TDP irradiation in the treatment of cold-damp type knee osteoarthritis. Xinjiang J Tradit Chin Med. 2015;33(2):17-8.
73. Liu YF, Lu JP. Clinical effect analysis of warming needle moxibustion in treatment of knee osteoarthritis. Continuing Med Edu. 2016;30(5):154-5.
74. Liu QL. Clinical observation on the therapeutic effect of warming acupuncture on knee osteoarthritis. Clin J Chin Med. 2016;8(09):108-9.
75. Liu QL. Clinical effect of warming acupuncture on knee osteoarthritis. Chin Foreign Med Res. 2017;15(18):121-3.
76. Liu M. Evaluation of the effect of warming acupuncture on knee osteoarthritis. J Clin Med Literature. 2017;4(63):12331-2.
77. Liu CH, Li Y. Comparison of the effect of warming acupuncture and general acupuncture on knee osteoarthritis of the yangxu hanning type. Clin J Chin Med. 2018;10(14):78-80.
78. Lu JJ, Ou YBS. Comparison of clinical effects between acusector and warm needling moxibustion in treating Knee osteoarthritis of blood Stasis obstruction pattern. West J Tradit Chin Med. 2014(4):119-21.
79. Ma W, Cao L, Lin L. Warm acupuncture combined with drug injection in the treatment of knee osteoarthritis Asia Pac Tradit Med. 2013;9(3):81-2.
80. Ma SJ, Yang K, Li FQ. Clinical observation on 54 cases of knee osteoarthritis treated with warm acupuncture and moxibustion. Zhejiang J Tradit Chin Med. 2015;50(10):751.
81. Ma SL. Clinical analysis of warm acupuncture combined with sodium hyaluronate injection in the treatment of osteoarthritis Shanxi J Tradit Chin Med. 2016;37(1):101-2.
82. Ma FC, Zhao L. Effects of warm acupuncture and moxibustion on CRP, ESR and joint function in the treatment of knee osteoarthritis Mod Med Health Res. 2020;4(1):115-6.
83. Ma MY, Zheng WN, Zeng HT. Effects of warm acupuncture combined with sodium hylauronate in treatment of patients with knee osteoarthritis. Med J Chin People Health. 2021;33:68-70.
84. Mei J, Yang ZR, Li H. Observation on therapeutic effect of warming acupuncture on knee osteoarthritis in early and middle stage. Shanghai J Acu-mox. 2014;33(1):51-3.
85. Mo Y. The clinical curative observation of needle warming moxibustion on knee osteoarthritis. Guangzhou: Guangzhou Univ Chin Med; 2015.
86. Niu SF, Zeng FG. Observation on therapeutic effect of warming acupuncture on knee osteoarthritis of deficiency of liver and kidney. Hainan Med J. 2014(18):2763-4.
87. Ou YBS, Gao J, Yang HZ. Comparison of the clinical effect of warm acupuncture and electroacupuncture on knee osteoarthritis of yang deficiency cold condensate type. Chin J Rehabil Med. 2011;26(3):265-7.
88. Ou L, Zhang L, Wang Q, Lu M. R CTs of needle-warming moxibustion and intra-articular injection in the treatment of knee ostarthritis. JCAM. 2018;34(1):8-11.
89. Pan L. Clinical observation of yang deficiency hanning type of knee osteoarthritis with the warm acupuncture and electro - acupuncture. Jinan: Shandong Univ Tradi Chin Med; 2014.
90. Pan S, Wang Q, Zhou X. Observation on the curative effect of warming acupuncture in the treatment of knee osteoarthritis. J General Practice. 2020;18:1362-4.
91. Peng L. Clinical Observation on Treatment of Senile Genual Osteoarthritis by Warm Needle Moxibustion. Liaoning J Tradit Chin Med. 2009;36(10):1773-4.
92. Qi YY, Cao S. Clinical study of warm acupuncture-moxibustion in the treatment of knee osteoarthritis (yang deficiency and cold coagulation type）. Cardiovas Dis Electr J Integr Tradi Chin West Med. 2017;5(34):169, 72.
93. Qiao H. Clinical effect of warming acupuncture and moxibustion on knee osteoarthritis. Special Health. 2018;12(24):252.
94. Qin Y. Treatment of 40 cases of senile knee osteoarthritis with warm acupuncture and moxibustion Chin J Geront. 2013;40(16):4029-31.
95. Qiu S. The study of the warm acupuncture treatment of knee osteoarthritis with randomized controlled methods. J Pracit Tradit Chin Intern Med. 2013;7(27):86-7.
96. Qiu DS, Zhang YH. Treatment of 34 cases of knee osteoarthritis with warm acupuncture. Shaanxi J Tradit Chin Med. 2013(10):1398-9.
97. Qu H, Wang R, Liu J. Clinical observation on warm needling in canicular days for knee osteoarthritis. J Acu Tuina Sci. 2015;13:63-6.
98. Ren J, Li T. Acupuncture combined with sodium hyaluronate in warming acupuncture of knee osteoarthriris efficacy. J Xinjiang Med Univ. 2012;35(9):1212-5.
99. Shi WH, Li L. Clinical observation of warm acupuncture combined with sodium hyaluronate in the treatment of early and middle stage knee osteoarthritis Shanxi J Tradit Chin Med. 2016;32(02):41-2.
100. Shu H. Observation on effect of articular injection of sodium hyaluronate combined with warm needle in treating knee osteoarthritis. World Chin Med. 2014(11):1528-30.
101. Shu Y. Observation on the effect of warm acupuncture and moxibustion in the treatment of knee osteoarthritis. Our Health. 2021;19:91-2.
102. Si QGW, Dong QM, Narenmandula. Treatment of 35 cases of knee osteoarthritis with mongolian medicine silver needle heating and sodium hyaluronate injection. Global Trad Chin Med. 2013;6(04):286-8.
103. Song CT. To observe the clinical efficacy and safety of warming needle moxibustion in the treatment of knee osteoarthritis. World Latest Med Inf. 2016;16(71):193-.
104. Su D, Song Y, Wang X, Liu J, Huang W. Observation of curative efficacy "shuanggu yitong" warm acupuncture and moxibustion in treatment of knee osteoarthritis and its effect on expression of serum inflammatory facotras. Shandong J Tradit Chin Med. 2020;39(4):378-81.
105. Sun Y, He L. Observation on the therapeutic effect of acupuncture and moxibustion combined with acupuncture and moxibustion on knee osteoarthritis. Clin J Tradit Chin Med. 2012;24(5):419-20.
106. Teng CG. Comparative observation on the clinical effect of warming acupuncture and electroacupuncture on knee osteoarthritis. Clin J Chin Med. 2012;4(12):34-5.
107. Teng JZ, Li ZH. Clinical observation of intensive silver needle thermotherapy combined with sodium hyaluronate intra-articular injection in the treatment of knee osteoarthritis Guangxi Med J. 2020;42(7):898-900.
108. Tian BQ. Observation on therapeutic effect of acupuncture and moxibustion on 120 cases of knee osteoarthritis. Proceedings of the First Academic Conference of Minimally Invasive Traditional Chinese Medicine of the Chinese Society of Traditional Chinese Medicine; Daqing2006. p. 494-6.
109. Tu XS, Liu XA, Hu LX. Clinically randomized controlled study of warm acupuncture in the treatment of knee osteoarthritis. JCAM. 2016;32(6):38-40.
110. Wang JG, He LJ. Observation on the therapeutic effect of warming needle moxibustion on knee osteoarthritis. Chin Acu-Mox. 2007;27(3):191-2.
111. Wang JF, Li X. Effect of warming needle moxibustion combined with sodium hyaluronate injection in knee joint for treatment of knee osteoarthritis with deficiency cold type Clin J Rehabil Med. 2010;25(11):1094-7.
112. Wang HF, Cheng SD, Li W, Xu HL. Randomly controlled trial of silver needles plus sodium hyaluronate for the treatment of knee osteoarthritis Shanghai J Acu-mox. 2011;30(4):250-1.
113. Wang F. Analysis of treatment effect of warming needle moxibustion on knee osteoarthritis of deficiency cold type. J Med Res. 2015;44(10):164-6.
114. Wang XR, Hong KD, Gan AF. Effect of knee osteoarthritis treated by warm needle moxibustion combined with glucosamine. Fujian J Tradit Chin Med. 2015;46(5):14-6.
115. Wang T, Cui XD. Observation on the effect of intra-articular injection of sodium hyaluronate combined with warm acupuncture on knee osteoarthritis. World Clin Med. 2015;9(6):140.
116. Wang L. Therapeutic effect of warm acupuncture on knee osteoarthritis Chin J Ethnomodicine Ethnopharmacy. 2016;25(8):73-4.
117. Wang MM, Cai SC. Clinical observation on warm needling with moxibustion in treating knee osteoarthritis due to yang-insufficiency and cold-congelation. Gansu Univ Chin Med. 2017;34(1):58-61.
118. Wang J. The clinical efficacy and safety studies of electric acupuncture and warm acupuncture in treatment of knee osteoarthritis of blood stasis obstruction type. Lab Med Clinic 2017;14(4):508-9.
119. Wang XL, Wang XB, Hou MJ, Wang HH, Ji F. Warm-needling moxibustion for knee osteoarthritis:a randomized controlled trial. Chin Acu-Mox. 2017;37(5):457-62.
120. Wang Y. Therapeutic effect of warm acupuncture and moxibustion combined with intra-articular injection of sodium hyaluronate in the treatment of knee osteoarthritis. J Electorcardiogram. 2018;7(2):198-9.
121. Wang ZQ, Zhao Y, Wang LH. Clinical observation of regular warming acupuncture and moxibustion in the treatment of knee osteoarthritis Chin J Tradit Med Sci Technol. 2019;26(2):307-8.
122. Wang D, Su YL. Effect of warm acupuncture combined with intra-articular injection of sodium hyaluronate on knee osteoarthritis. Chin Reflexolocy 2019;217(11):19-20.
123. Wei YZ. Warm acupuncture combined with sodium hyaluronate intra-articular injection in the treatment of 40 cases of knee osteoarthritis. J External Therapy TCM. 2013;22(5):16-7.
124. Wu YL. Therapeutic effect of warm acupuncture on 60 cases of knee osteoarthritis New J Tradit Chin Med. 2006;1(38):66-7.
125. Wu Y ZJ, Li S. . Clinical observation on treatment of knee osteoarthritis by warm needling method. J Acu Tuina Sci. 2009;7:3349-351.
126. Wu Y, Zhang Y, Tang C. Effect of moxibustion with warming needling on IL-17 and IL-18 of knee osteoarthritis patients. Liaoning J Tradit Chin Med. 2013;40(5):864-6.
127. Wu SY. The observation on therapeutic effect of warming acupuncture-moxibustion for knee osteoarthritis of syndrome of yang deficiency and coagulated cold. Guangzhou: Guangzhou Univ Chin Med; 2013.
128. Wu CH. Clinical study of warming acupuncture and moxibustion in the treatment of knee osteoarthritis. Zhengzhou: Henan Univ Tradi Chin Med; 2016.
129. Wu ZY. Observation on therapeutic effect of warm acupuncture combined with sodium hyaluronate on knee osteoarthritis. J Clin Med Literature. 2017;4(33):6467-8.
130. Wu GM. Effect of warming needle moxibustion combined with hyaluronic acid injection on osteoarthritis and its influence on inflammatory factors CJGMCM. 2018;33(18):2716-8.
131. Xia DB, Huang WM, Wang XL, Huang Y. Clinical observation of needle warming moxibustion combined with cluster acusector on patients with knee osteoarthritis. J Tradit Chin Med Univ Hunan. 2012;32(11):74-7.
132. Xu HB. Application of warm acupuncture in the treatment of knee osteoarthritis in the elderly. For all Health. 2014;8(08):41.
133. Xu X, Wu MX. Clinical observation of warming needling on patients with knee osteoarthritis in Botswana Chin Man-Rehabil Med. 2019;10(19):41-2.
134. Xu HB, Zeng Y. Therapeutic effect of warm acupuncture on knee osteoarthritis J Pract Tradit Chin Med. 2020;36(8):1087-8.
135. Xue L. Clinical observation on knee osteoarthritis treated by acupuncture with moxibuation: Hubei Univ Chin Med; 2011.
136. Xue PH, Qu CZ, Peng XM. Clinical observation of warming acupuncture and moxibustion in the treatment of knee osteoarthritis Chin Man-Rehabil Med. 2015;6(11):32-3.
137. Yang XC, He SF, Wang RC, Zhou YM. Observation on curative effect of thermal acupuncture needle muscular stimulation therapy for knee osteoarthritis patients. Acu Res. 2012;37(3):237-41.
138. Yang D. Clinical study on the treatment of degenerative knee osteoarthritis (yang deficiency and cold coagulation). For all Health. 2014;8(2):47-8.
139. Yang WC. Clinical curative effect observation of warm needle moxibustion treatment knee osteoarthritis with kidney-yang deficiency and cold type J Chin Prescr Drug. 2015(2):14-6.
140. Yang KY, Qu XX. Clinical observation on the treatment of knee osteoarthritis with warm acupuncture. Shaanxi Journal of Traditional Chinese Medicine. 2015(11):1520-1.
141. Yao XB. Randomized controlled study of warming acupuncture and moxibustion in the treatment of knee osteoarthritis. Inner Mongolia J Tradit Chin Med. 2018;37(6):83-4.
142. Ying HZ, Huang F, Ying HF. Effect of needle warming moxibustion and electroacupuncture in treatment of knee osteoarthritis. J Zhejiang Chin Med Univ. 2015(2):150-2.
143. Yu J. Observation on 32 cases of knee osteoarthritis treated by warm acupuncture and moxibustion. J Pract Tradit Chin Med. 2009;25(2):93.
144. Yu M. Clinical observation on 40 cases of knee osteoarthritis treated by knee four needle warm acupuncture and moxibustion Chin Naturop. 2016;24(4):25.
145. Yu XC, Lin SS. Effect of sodium hyaluronate combined with warm needling on knee osteoarthritis Shanxi J Tradit Chin Med. 2016;32(4):30-2.
146. Zeng Y, Xuan TH. Observation on therapeutic effect of warming acupuncture on knee osteoarthritis. Mod Diagn Treatment. 2016;27(4):633-4.
147. Zeng ZW. Clinical observation of sodium hyaluronate combined with acupuncture in the treatment of knee osteoarthritis. Mod Diagn Treatment. 2017;28(9):1599-600.

148. Zhang JF, Wu YC, Li SS. Observation on therapeutic effect of penetrating needling and warming acupuncture on knee osteoarthritis. Shanghai J Acu-mox. 2009;28(12):722-3.
149. Zhang YS, Wang ZX. Randomized controlled clinical trails for treatment of knee osteoarthritis by warm acupuncture combined with Intra-articular injection of sodium hyaluronate. Acu Res. 2011;36(5):373-6.
150. Zhang HC, Wen LP, Wu QM. 30 cases of knee osteoarthritis with deficiency of liver and kidney treated by warming acupuncture and electroacupuncture. Hunan J Tradit Chin Med. 2013;29(05):85-7.
151. Zhang J. 90 cases of knee osteoarthritis treated with warm acupuncture and moxibustion. Chin Med Modern Distance Educ Chin. 2013;11(22):77-.
152. Zhang YX. Clinic effect comparison of electroacupuncture and moxibustion for knee osteoarthritis of kidney and marrow deficiency. Med Recapitulate. 2013;19(10):1903-5.
153. Zhang Y. The clinical efficacy of warming moxibustion treatment of knee osteoarthritis research and related cytokines. Ningxia: Ningxia Med Univ; 2014.
154. Zhang J. Curative effect observation of warm needle moxibustion treatment knee osteoarthritis(KOA)with kidney-yang deficiency and dold type. China Health Stand Manag. 2016;7(12):128-9.
155. Zhang XR, Lu WX. Observation on the short-term therapeutic effect of warming acupuncture on knee osteoarthritis. Chin Med Clin Res 2016;8(05):15-6.
156. Zhang BX. Therapeutic effect of warming acupuncture on early and middle stage knee osteoarthritis. World J Complex Med. 2016;2(2):38-41.
157. Zhang BA. Clinical effect of warm acupuncture and moxibustion in treatment of osteorthritis of the knee joint. Chin Reflexolocy 2017;159(1):14-5.
158. Zhang HM, Chen YC, He JQ. Clinical obaservation on treating mild to moderate knee osteoarthritis with warm needling combined with intra-articular injection of sodium hyaluronate. Rheum Arthritis. 2018;7(5):22-4, 47.
159. Zhang C, Jiao Z. Effect of warm acupuncture and moxibustion on pain and knee joint motor function in patients with knee osteoarthritis. CJGMCM. 2019;34(13):2042-4.
160. Zhang PL, Zhang JF, Huang K, Zhu SY, Wan JM. Effect of warm needling moxibustion plus loxoprofen sodium on bone metablism indicators and serum IL-6, IL-1 and SOD in elderly patients with acute exacerbation of knee osteoarthritis. Shanghai J Acu-mox. 2019;38(9):1043-7.
161. Zheng ZJ. Observation on the therapeutic effect of warm acupuncture and Yang needling on knee osteoarthritis JCAM. 2008;24(2):30-1.
162. Zheng CJ. Clinical observation on 38 cases of knee osteoarthritis treated with integrated traditional Chinese and Western Medicine Chin J Ethnomodicine Ethnopharmacy. 2016;25(18):95-6,9.
163. Zhong GC, Chen ZH. Clinical observation on 30 cases of knee osteoarthritis treated with warm acupuncture and moxibustion. Jiangsu J Tradit Chin Med. 2008;40(10):79-80.
164. Zhou WN, Wang TM. 54 cases of degenerative knee arthritis treated by warming acupuncture and moxibustion. J Clin Acu Mox. 2006(11):31.
165. Zhou WC. Cli nical comparative study of electro-acupuncture and acupuncture debate temperature on kidney marrow deficiency syndrome of knee osteoarthritis. J Emerg Tradit Chin Med. 2014;23(8):1521-3.
166. Zhou WJ, Zhang W, Lou BD. Therapeutic effect of warming acupuncture on knee osteoarthritis of yang deficiency and cold coagulation. Hunan J Tradit Chin Med. 2014;30(4):90-3.
167. Zhou M. Observation on therapeutic effect of acupuncture and moxibustion on knee osteoarthritis. Asia Pac Tradit Med. 2015;11(18):99-100.
168. Zhou HL. Clinical observation of warm acupuncture combined with intra-articular injection of sodium hyaluronate in the treatment of knee osteoarthritis. Capital Med. 2020(7):197.
169. Zhu L, Jin Z, Liao LQ, Xu LK, lI Y. Clinical Study on the Treatment of 40 Cases of Early Senile Knee Osteoarthritis with Warm Acupuncture and Moxibustion. Chin Community Doctors. 2011;13(21):190-1.
170. Zhu JL, Gu JH. Clinical effect of warm acupuncture on knee osteoarthritis. J Tradit Chin Med Neimenggu. 2017;36(04):109.
171. Zuo Z, Jiang YW. Clinical observation on moxibustional-warmed needling for the non effusion osteoarthritis in the knees. J Yunnan Univ Tradit Chin Med. 2011;34(5):49-51.
172. Zuo C. Effect of warm acupuncture and moxibustion on free radical metabolism in patients with knee osteoarthritis JCAM. 2015;31(10):49-51.

Supplementary 4. Quality evaluation using AMSTAR 2 checklist

**First author year**	**Q1**	**Q2**	**Q3**	**Q4**	**Q5**	**Q6**	**Q7**	**Q8**	**Q9**	**Q10**	**Q11**	**Q12**	**Q13**	**Q14**	**Q15**	**Q16**	**Rating overall confidence***	**Number of No**
Feng 2019	**Y**	**N**	**N**	**PY**	**Y**	**Y**	**N**	**PY**	**Y**	**Y**	**Y**	**N**	N	**Y**	**Y**	**N**	**CL**	**6**
Guo 2018	**Y**	**N**	**N**	**PY**	**Y**	**N**	**N**	**PY**	**Y**	**N**	**Y**	**N**	**N**	**Y**	**Y**	**N**	**CL**	**8**
Lu 2015	**Y**	**N**	**N**	**PY**	**N**	**N**	**N**	**PY**	**Y**	**N**	**Y**	**N**	**N**	**Y**	**Y**	**N**	**CL**	**9**
Kong 2019	**Y**	**N**	**Y**	**Y**	**Y**	**Y**	**N**	**PY**	**Y**	**N**	**Y**	**Y**	**Y**	**Y**	**Y**	**N**	**L**	**4**
Cao 2019	**Y**	**N**	**N**	**PY**	**Y**	**Y**	**N**	**PY**	**Y**	**Y**	**Y**	**N**	**N**	**Y**	**Y**	**N**	**CL**	**6**
Jun 2022	**Y**	**Y**	**Y**	**Y**	**Y**	**Y**	**N**	**Y**	**Y**	**Y**	**Y**	**Y**	**Y**	**Y**	**Y**	**Y**	**M**	**1**
Chen 2019	**Y**	**N**	**N**	**Y**	**Y**	**Y**	**N**	**Y**	**N**	**N**	**Y**	**Y**	**N**	**N**	**Y**	**N**	**CL**	**8**
Jiang 2019	**Y**	**N**	**Y**	**Y**	**Y**	**Y**	**N**	**PY**	**Y**	**N**	**Y**	Y	N	Y	**Y**	**N**	**CL**	**5**
Huang 2021	**Y**	**N**	**Y**	**PY**	**Y**	**Y**	**N**	**Y**	**Y**	**Y**	**Y**	**Y**	**N**	**Y**	**Y**	**N**	**L**	**4**
Luo 2019	**Y**	**N**	**Y**	**PY**	**Y**	**N**	**N**	**PY**	**Y**	**Y**	**Y**	**N**	**Y**	**Y**	**Y**	**N**	**CL**	**5**
Jin 2022	**Y**	**N**	**Y**	**Y**	**Y**	**Y**	**N**	**PY**	**Y**	**Y**	**Y**	**N**	**N**	**Y**	**Y**	**Y**	**L**	**4**
Li 2021	**Y**	**N**	**Y**	**Y**	**Y**	**Y**	**N**	**Y**	**Y**	**Y**	**Y**	**Y**	**Y**	**Y**	**Y**	**N**	**L**	**3**
Zhang 2018	**Y**	**N**	**Y**	**PY**	**N**	**Y**	**N**	**PY**	**Y**	**N**	**Y**	**N**	**Y**	**Y**	**Y**	**N**	**CL**	**6**
Ou 2018	**Y**	**N**	**N**	**PY**	**Y**	**Y**	**N**	**PY**	**Y**	**Y**	**Y**	**N**	**Y**	**Y**	**Y**	**N**	**CL**	**5**
Wu 2016	**Y**	**N**	**N**	**PY**	**Y**	**Y**	**N**	**N**	**N**	**Y**	**Y**	**N**	**N**	**Y**	**Y**	**N**	**CL**	**8**

Y: yes; PY: partial yes; N: no
Note:
*AMSTAR2 was used to critically appraise the reporting quality of each included SR. The overall confidence of each SR was graded as "high" (no or non-critical weakness in all items), "moderate" (more than one non-critical weakness among all the items), "low" (one critical flaw with or without non-critical weakness), or "critically low" (more than one critical flaw with or without non-critical weakness).
Q1: Did the research questions and inclusion criteria for the review included the components of PICO?
Q2: Did the report of the review contain an explicit statement that the review methods were established prior to the conduct of the review and did the report justify any significant deviations from the protocol?
Q3: Did the review authors explain their selection of the study designs for inclusion in the review?
Q4. Did the review authors use a comprehensive literature search strategy?
Q5: Did the review authors perform study selectin in duplicate?
Q6: Did the review authors perform data extraction in duplicate?
Q7: Did the review authors provide a list of excluded studies and justify the exclusions?
Q8: Did the review authors describe the included studies in adequate detail?
Q9: Did the review authors used a satisfactory technique for assessing the risk of bias (ROB) in individual studies that were included in the review?
Q10: Did the review authors report on the sources of funding for the studies included in the review?
Q11: If meta-analysis was performed did the review authors use appropriate methods for statistical combination of results?
Q12: If meta-analysis was performed, did the review authors assess the potential impact of ROB in individual studies on the results of the meta-analysis or other evidence synthesis?
Q13: Did the review authors account for ROB in individual studies when interpreting/discussing the results of the review?
Q14: Did the review authors provide a satisfactory explanation for, and discussion of, any heterogeneity observed in the results of the review?
Q15: If they performed quantitative synthesis did the review authors carry out an adequate investigation of publication bias (small study bias) and discuss its likely impact on the results of the review?
Q16: Did the review authors report any potential sources of conflict of interest, including any funding they received for conducting the review?

*Evaluated rating overall confidence by the number of No: 0 = H / 1 = M / 2~4 = L / >5 = CL

Supplementary 5. PRISMA 2020 checklist

First author year	Q1	Q2	Q3	Q4	Q5	Q6	Q7	Q8	Q9	Q10	Q11	Q12	Q13	Q14	Q15	Q16	Q17	Q18	Q19	Q20	Q21	Q22	Q23	Q24	Q25	Q26	Q27
Feng 2019	Y	Y	Y	Y	N	PY	Y	Y	Y	Y	Y	Y	N	Y	N	PY	PY	Y	Y	Y	Y	N	PY	N	Y	N	N
Guo 2018	Y	Y	PY	Y	N	PY	PY	Y	Y	Y	Y	Y	Y	Y	N	PY	Y	Y	Y	Y	Y	N	PY	N	N	N	N
Lu 2015	Y	Y	Y	Y	N	PY	Y	Y	N	N	Y	N	PY	N	N	PY	Y	Y	Y	Y	Y	N	Y	N	N	N	N
Kong 2019	Y	Y	Y	Y	Y	Y	Y	Y	Y	Y	Y	Y	Y	Y	N	Y	Y	Y	Y	Y	Y	N	Y	N	N	N	N
Cao 2019	Y	Y	Y	Y	N	PY	Y	Y	Y	N	Y	Y	Y	Y	N	PY	PY	Y	Y	Y	Y	N	Y	N	Y	N	N
Jun 2022	Y	Y	Y	Y	Y	Y	Y	Y	Y	Y	Y	Y	Y	Y	Y	Y	Y	Y	Y	Y	Y	Y	Y	Y	Y	Y	Y
Chen 2019	Y	Y	Y	Y	N	Y	PY	Y	Y	N	N	N	N	Y	N	PY	Y	N	Y	Y	Y	N	PY	N	N	N	N
Jiang 2019	Y	Y	Y	Y	Y	Y	PY	Y	Y	Y	Y	Y	Y	Y	N	Y	PY	Y	Y	Y	Y	N	Y	N	N	N	N
Huang 2021	Y	Y	Y	Y	Y	PY	Y	Y	Y	Y	Y	Y	Y	Y	N	Y	PY	Y	Y	Y	Y	N	Y	N	Y	N	N
Luo 2019	Y	Y	PY	Y	Y	PY	PY	Y	Y	N	Y	N	Y	Y	N	Y	PY	Y	Y	Y	Y	N	PY	N	Y	N	N
Jin 2022	Y	Y	Y	Y	Y	Y	PY	Y	Y	Y	Y	Y	Y	Y	N	Y	Y	Y	Y	Y	Y	N	Y	N	Y	Y	Y
Li 2021	Y	Y	Y	Y	Y	Y	PY	Y	Y	Y	Y	Y	Y	Y	N	Y	Y	Y	Y	Y	Y	N	Y	N	Y	N	N
Zhang 2018	Y	Y	Y	Y	Y	PY	Y	Y	N	Y	Y	Y	Y	N	N	Y	PY	Y	Y	Y	Y	N	Y	N	N	N	N
Ou 2018	Y	Y	PY	Y	N	PY	Y	Y	N	N	Y	N	Y	N	N	PY	PY	Y	Y	Y	Y	N	PY	N	Y	N	N
Wu 2016	Y	Y	Y	Y	N	PY	Y	Y	Y	N	PY	N	N	Y	N	PY	PY	Y	Y	Y	Y	N	PY	N	Y	N	N

Y: yes; PY: partial yes; N: no

Q1. Title: Identify the report as a systematic review.

Q2. Abstract: See the PRISMA 2020 for Abstracts checklist.

Q3. Rationale: Describe the rationale for the review in the context of existing knowledge.

Q4. Objectives: Provide an explicit statement of the objective(s) or question(s) the review addresses.

Q5. Eligibility criteria: Specify the inclusion and exclusion criteria for the review and how studies were grouped for the syntheses.

Q6. Information sources: Specify all databases, registers, websites, organizations, reference lists and other sources searched or consulted to identify studies. Specify the date when each source was last searched or consulted.

Q7. Search strategy: Present the full search strategies for all databases, registers and websites, including any filters and limits used.

Q8. Selection process: Specify the methods used to decide whether a study met the inclusion criteria of the review, including how many reviewers screened each record and each report retrieved, whether they worked independently, and if applicable, details of automation tools used in the process.

Q9. Data collection process: Specify the methods used to collect data from reports, including how many reviewers collected data from each report, whether they worked independently, any processes for obtaining or confirming data from study investigators, and if applicable, details of automation tools used in the process.

Q10. Data items: 10a: List and defined all outcomes for which data were sought. Specify whether all results that were compatible with compatible with each outcome domain in each study were sought, and if not, the methods used to decide which results to collect. 10b: List and defined all other variables for which data were sought. Describe any assumptions made about any missing or unclear information.

Q11. Study risk of bias assessment: Specify the methods used to assessed risk of bias in the included studies, including details of the tool used, how many reviewers assessed each study and whether the they worked independently, and if applicable, details of automation tools used in the process.

Q12. Effect measures: Describe the rationale for the review in the context of existing knowledge.

Q13. Synthesis methods: 13a: Describe the processes used to decide which studies were eligible for each synthesis (e.g. tabulating the study intervention characteristics and comparing against the planned groups for each synthesis (item #5)). 13b: Describe any methods required to prepare the data for presentation or synthesis, such as handling of missing summary statistics, or data conversions. 13c: Describe any methods used to tabulate or visually display results of individual studies and syntheses. 13d: Describe any methods used to synthesize results and provide a rationale for the choice(s). If meta-analysis was performed, describe the model(s), method(s) to identify the presence and extent of statistical heterogeneity, and software package(s) used. 13e: Describe any methods used to explore possible causes of heterogeneity among study results (e.g. subgroup analysis, meta-regression). 13f: Describe any sensitivity analyses conducted to assess robustness of the synthesized results.

Q14. Reporting bias assessment: Describe any methods used to assess risk of bias due to missing results in a synthesis (arising from reporting biases).

Q15. Certainty assessment: Describe any methods used to assess certainty (or confidence) in the body of evidence for an outcome.

Q16. Study selection: 16a: Describe the results of the search and selection process, from the number of records identified in the search to the number of studies included in the review, ideally using a flow diagram. 16b: Cite studies that might appear to meet the inclusion criteria, but which were excluded, and explain why they were excluded.

Q17. Study characteristics: Cite each included study and present its characteristics.

Q18. Risk of bias in studies: Present assessments of risk of bias for each included study.

Q19. Results of individual studies: For all outcomes, present, for each study: (a) summary statistics for each group (where appropriate) and (b) an effect estimate and its precision (e.g. confidence/credible interval), ideally using structured tables or plots.

Q20. Results of syntheses: 20a: For each synthesis, briefly summarise the characteristics and risk of bias among contributing studies. 20b: Present results of all statistical syntheses conducted. If meta-analysis was done, present for each the summary estimate and its precision (e.g. confidence/credible interval) and measures of statistical heterogeneity. If comparing groups, describe the direction of the effect. 20c: Present results of all investigations of possible causes of heterogeneity among study results. 20d: Present results of all sensitivity analyses conducted to assess the robustness of the synthesized results.

Q21. Reporting biases: Present assessments of risk of bias due to missing results (arising from reporting biases) for each synthesis assessed.

Q22. Certainty of evidence: Present assessments of certainty (or confidence) in the body of evidence for each outcome assessed

Q23. Discussion: 23a: Provide a general interpretation of the results in the context of other evidence. 23b: Discuss any limitations of the evidence included in the review. 23c: Discuss any limitations of the review processes used. 23d: Discuss implications of the results for practice, policy, and future research.

Q24. Registration and protocol: 24a: Provide registration information for the review, including register name and registration number, or state that the review was not registered. 24b: Indicate where the review protocol can be accessed, or state that a protocol was not prepared. 24c: Describe and explain any amendments to information provided at registration or in the protocol.

Q25. Support: Describe sources of financial or non-financial support for the review, and the role of the funders or sponsors in the review.

Q26. Competing interests: Declare any competing interests of review authors.

Q27. Availability of data, code and other materials: Report which of the following are publicly available and where they can be found: template data collection forms; data extracted from included studies; data used for all analyses; analytic code; any other materials used in the review.

Supplement 6. Quality of evidence in included systematic reviews with GRADE

First author year	Outcomes	Certainty assessment					Quality of evidence*
		Risk of bias	Inconsistency	Indirectness	Imprecision	Publication bias	
Feng 2019	Total effective rate	-2	0	0	0	0	Low
Guo 2018	Total effective rate	-2	0	0	0	0	Low
	WOMAC total	-1	-2	0	0	0	Very low
Lu 2015	Total effective rate	-2	-2	0	0	0	Very low
	Total effective rate _ long term	-2	0	0	-1	0	Very low
	Total effective rate _ sort term	-1	0	0	-1	0	Low
	Adverse reactions	-1	0	0	-1	0	Low
Kong 2019	Total effective rate	-1	0	0	0	0	Moderate
	VAS	-2	-2	0	0	0	Very low
	LKSS	-2	-2	0	0	0	Very low
	WOMAC total	-2	-2	0	-1	0	Very low
Cao 2019	Total effective rate	-1	0	0	0	0	Moderate
Jun 2022	Total effective rate (WA vs. Drug)	-2	0	0	0	0	Low
	Total effective rate (WA vs. Injection)	-2	0	0	0	0	Very low
	Total effective rate (WA + Drug vs. Drug)	-2	0	0	0	0	Low
	Total effective rate (WA + Injection vs. Injection)	-2	-1	0	0	0	Very low
	Pain (WA vs. WM (Drug)	-2	-2	0	0	0	Very low
	Pain (WA vs. Injection)	-2	-2	0	0	0	Very low
	Pain (WA + Drug vs. Drug)	-2	-2	0	0	0	Very low
	Pain (WA + Injection vs. Injection)	-2	-1	0	0	0	Very low
	Function (WA vs. WM (Drug)	-2	-2	-1	0	0	Very low
	Function (WA vs. Injection)	-2	-2	-1	0	0	Very low
	Function (WA + Drug vs. Drug)	-2	-2	-1	0	0	Very low
	Function (WA + Injection vs. Injection)	-2	-1	0	0	0	Very low
Jiang 2019	Total effective rate	-1	0	0	0	0	Moderate
Chen 2019	VAS	-2	-2	0	0	0	Very low
	WOMAC total	-2	-1	0	0	0	Very low
Huang 2021	Total effective rate	-1	0	0	0	0	Moderate
	VAS	-1	-1	0	0	0	Low
	VAS	-1	-1	0	-1	0	Very low
	VAS	-1	0	0	-1	0	Low
	VAS	-1	0	0	-1	0	Low
	VAS	-1	0	0	-1	0	Low
	WOMAC total	-1	-1	0	0	0	Low
	WOMAC total	-1	-1	0	-1	0	Very low
	WOMAC total	-1	-2	0	-2	0	Very low
	WOMAC total	-1	0	0	-1	0	Low
Luo 2019	Total effective rate	-2	0	0	0	0	Low
Jin 2022	Total effective rate	-1	-1	0	0	0	Low
	Daily activities	-1	-1	0	-1	0	Low
	VAS	-1	-1	0	0	0	Very low
	WOMAC total	-1	-2	0	0	0	Low
Li 2021	Total effective rate	-1	0	0	0	0	Moderate
	Total effective rate	-1	0	0	-1	0	Low
	VAS	-1	0	0	-1	0	Low
	VAS	-1	-2	0	-1	0	Very low
	WOMAC total	-1	0	0	-2	0	Very low
	Legnesene	-1	-1	0	-1	0	Very low
	LKSS	-1	0	0	-1	0	Low
Zhang 2018	Total effective rate	-1	-1	0	-1	0	Very low
	VAS	-1	-2	0	-2	0	Very low
	LKSS	-1	-1	0	0	0	Low
	WOMAC	-1	0	0	-2	0	Very low
Ou 2018	Total effective rate	-2	0	0	0	0	Low
Wu 2016	Total effective rate	-1	0	0	-2	0	Very low
	Cure rate	-1	0	0	-2	0	Very low
	VAS	-1	-1	0	-2	0	Very low
	WOMAC	-1	0	0	-2	0	Very low

AT: acupuncture; GADE: Grades of Recommendations, Assessment, Development, and Evaluation; IA: intra-articular injection; LKSS: Lysholm score; VAS: visual analog scale; WA: warm needle acupuncture; WM: western medicine; WOMAC: Western Ontario and McMaster Universities Osteoarthritis index.

*reviewer's judgment, -2: very serious; -1: serious; 0: not serious

PRIOR Checklist

(Gates M, Gates A, Pieper D, et al. Reporting guideline for overviews of reviews of healthcare interventions: development of the PRIOR statement. *BMJ* 2022;378:e070849. doi:10.1136/bmj-2022-070849.)

**Section** Topic	**#**	**Item**	**Location reported**
**TITLE**			
Title	1	Identify the report as an overview of reviews.	page 1
**ABSTRACT**			
Abstract	2	Provide a comprehensive and accurate summary of the purpose, methods, and results of the overview of reviews.	page 1
**INTRODUCTION**			
Rationale	3	Describe the rationale for conducting the overview of reviews in the context of existing knowledge.	page 2
Objectives	4	Provide an explicit statement of the objective(s) or question(s) addressed by the overview of reviews.	page 3
**METHODS**			
Eligibility criteria	5a	Specify the inclusion and exclusion criteria for the overview of reviews. If supplemental primary studies were included, this should be stated, with a rationale.	page 3-4
	5b	Specify the definition of 'systematic review' as used in the inclusion criteria for the overview of reviews.	page 3
Information sources	6	Specify all databases, registers, websites, organizations, reference lists, and other sources searched or consulted to identify systematic reviews and supplemental primary studies (if included). Specify the date when each source was last searched or consulted.	page 3
Search strategy	7	Present the full search strategies for all databases, registers and websites, such that they could be reproduced. Describe any search filters and limits applied.	page 3 supplementary file
Selection process	8a	Describe the methods used to decide whether a systematic review or supplemental primary study (if included) met the inclusion criteria of the overview of reviews.	page 4
	8b	Describe how overlap in the populations, interventions, comparators, and/or outcomes of systematic reviews was identified and managed during study selection.	page 4-5
Data collection process	9a	Describe the methods used to collect data from reports.	page 4
	9b	If applicable, describe the methods used to identify and manage primary study overlap at the level of the comparison and outcome during data collection. For each outcome, specify the method used to illustrate and/or quantify the degree of primary study overlap across systematic reviews.	page 4-5
	9c	If applicable, specify the methods used to manage discrepant data across systematic reviews during data collection.	page 4
Data items	10	List and define all variables and outcomes for which data were sought. Describe any assumptions made and/or measures taken to identify and clarify missing or unclear information.	Table 1
Risk of bias assessment	11a	Describe the methods used to *assess* risk of bias or methodological quality of the included systematic reviews.	page 4-5
	11b	Describe the methods used to *collect* data on (from the systematic reviews) and/or *assess* the risk of bias of the primary studies included in the systematic reviews. Provide a justification for instances where flawed, incomplete, or missing assessments are identified but not re-assessed.	page 5
	11c	Describe the methods used to *assess* the risk of bias of supplemental primary studies (if included).	page 5
Synthesis methods	12a	Describe the methods used to summarize or synthesize results and provide a rationale for the choice(s).	page 6
	12b	Describe any methods used to explore possible causes of heterogeneity among results.	none
	12c	Describe any sensitivity analyses conducted to assess the robustness of the synthesized results.	none
Reporting bias assessment	13	Describe the methods used to *collect* data on (from the systematic reviews) and/or *assess* the risk of bias due to missing results in a summary or synthesis (arising from reporting biases at the levels of the systematic reviews, primary studies, and supplemental primary studies, if included).	page 5
Certainty assessment	14	Describe the methods used to *collect* data on (from the systematic reviews) and/or *assess* certainty (or confidence) in the body of evidence for an outcome.	page 5-6
**RESULTS**			
Systematic review and supplemental primary study selection	15a	Describe the results of the search and selection process, including the number of records screened, assessed for eligibility, and included in the overview of reviews, ideally with a flow diagram.	page 7
	15b	Provide a list of studies that might appear to meet the inclusion criteria, but were excluded, with the main reason for exclusion.	page 7

**Section** Topic	**#**	**Item**	**Location reported**
Characteristics of systematic reviews and supplemental primary studies	16	Cite each included systematic review and supplemental primary study (if included) and present its characteristics.	page 7
Primary study overlap	17	Describe the extent of primary study overlap across the included systematic reviews.	page 8
Risk of bias in systematic reviews, primary studies, and supplemental primary studies	18a	Present assessments of risk of bias or methodological quality for each included systematic review.	page 10
	18b	Present assessments (*collected* from systematic reviews or *assessed* anew) of the risk of bias of the primary studies included in the systematic reviews.	page 10
	18c	Present assessments of the risk of bias of supplemental primary studies (if included).	supplementary file page 10
Summary or synthesis of results	19a	For all outcomes, summarize the evidence from the systematic reviews and supplemental primary studies (if included). If meta-analyses were done, present for each the summary estimate and its precision and measures of statistical heterogeneity. If comparing groups, describe the direction of the effect.	Table 2
	19b	If meta-analyses were done, present results of all investigations of possible causes of heterogeneity.	none
	19c	If meta-analyses were done, present results of all sensitivity analyses conducted to assess the robustness of synthesized results.	none
Reporting biases	20	Present assessments (*collected* from systematic reviews and/or *assessed* anew) of the risk of bias due to missing primary studies, analyses, or results in a summary or synthesis (arising from reporting biases at the levels of the systematic reviews, primary studies, and supplemental primary studies, if included) for each summary or synthesis assessed.	page 10-11 supplementary file
Certainty of evidence	21	Present assessments (*collected* or *assessed* anew) of certainty (or confidence) in the body of evidence for each outcome.	page 11
**DISCUSSION**			
Discussion	22a	Summarize the main findings, including any discrepancies in findings across the included systematic reviews and supplemental primary studies (if included).	page 11
	22b	Provide a general interpretation of the results in the context of other evidence.	page 11-12
	22c	Discuss any limitations of the evidence from systematic reviews, their primary studies, and supplemental primary studies (if included) included in the overview of reviews. Discuss any limitations of the overview of reviews methods used.	page 12-13
	22d	Discuss implications for practice, policy, and future research (both systematic reviews and primary research). Consider the relevance of the findings to the end users of the overview of reviews, e.g., healthcare providers, policymakers, patients, among others.	page 13
**OTHER INFORMATION**			
Registration and protocol	23a	Provide registration information for the overview of reviews, including register name and registration number, or state that the overview of reviews was not registered.	page 3
	23b	Indicate where the overview of reviews protocol can be accessed, or state that a protocol was not prepared.	page 3
	23c	Describe and explain any amendments to information provided at registration or in the protocol. Indicate the stage of the overview of reviews at which amendments were made.	none
Support	24	Describe sources of financial or non-financial support for the overview of reviews, and the role of the funders or sponsors in the overview of reviews.	page 14
Competing interests	25	Declare any competing interests of the overview of reviews' authors.	page 14
Author information	26a	Provide contact information for the corresponding author.	page1, 14
	26b	Describe the contributions of individual authors and identify the guarantor of the overview of reviews.	page 14
Availability of data and other materials	27	Report which of the following are available, where they can be found, and under which conditions they may be accessed: template data collection forms; data collected from included systematic reviews and supplemental primary studies; analytic code; any other materials used in the overview of reviews.	supplementary files
